# NG2, a common denominator for neuroinflammation, blood–brain barrier alteration, and oligodendrocyte precursor response in EAE, plays a role in dendritic cell activation

**DOI:** 10.1007/s00401-016-1563-z

**Published:** 2016-03-30

**Authors:** Giovanni Ferrara, Mariella Errede, Francesco Girolamo, Sara Morando, Federico Ivaldi, Nicolò Panini, Caterina Bendotti, Roberto Perris, Roberto Furlan, Daniela Virgintino, Nicole Kerlero de Rosbo, Antonio Uccelli

**Affiliations:** Department of Neurology, Rehabilitation, Ophthalmology, Genetics, Maternal and Child Health, University of Genoa, Genoa, Italy; Department of Basic Medical Sciences, Neurosciences and Sense Organs, University of Bari, Bari, Italy; Department of Neuroscience, IRCCS-Mario Negri Institute for Pharmacological Research, Milan, Italy; Centre for Molecular and Translational Oncology, University of Parma, Parma, Italy; Division of Neuroscience, Institute of Experimental Neurology, San Raffaele Scientific Institute, Milan, Italy; Centre of Excellence for Biomedical Research, University of Genoa, Genoa, Italy; IRCCS AOU San Martino – IST, Genoa, Italy

**Keywords:** Nerve/glial-antigen 2, Experimental autoimmune encephalomyelitis, Oligodendrocyte precursor cells, Blood–brain barrier, Tight junctions, DC activation

## Abstract

In adult CNS, nerve/glial-antigen 2 (NG2) is expressed by oligodendrocyte progenitor cells (OPCs) and is an early marker of pericyte activation in pathological conditions. NG2 could, therefore, play a role in experimental autoimmune encephalomyelitis (EAE), a disease associated with increased blood–brain barrier (BBB) permeability, inflammatory infiltrates, and CNS damage. We induced EAE in NG2 knock-out (NG2KO) mice and used laser confocal microscopy immunofluorescence and morphometry to dissect the effect of NG2 KO on CNS pathology. NG2KO mice developed milder EAE than their wild-type (WT) counterparts, with less intense neuropathology associated with a significant improvement in BBB stability. In contrast to WT mice, OPC numbers did not change in NG2KO mice during EAE. Through FACS and confocal microscopy, we found that NG2 was also expressed by immune cells, including T cells, macrophages, and dendritic cells (DCs). Assessment of recall T cell responses to the encephalitogen by proliferation assays and ELISA showed that, while WT and NG2KO T cells proliferated equally to the encephalitogenic peptide MOG35-55, NG2KO T cells were skewed towards a Th2-type response. Because DCs could be responsible for this effect, we assessed their expression of IL-12 by PCR and intracellular FACS. IL-12-expressing CD11c+ cells were significantly decreased in MOG35-55-primed NG2KO lymph node cells. Importantly, in WT mice, the proportion of IL-12-expressing cells was significantly lower in CD11c+ NG2- cells than in CD11c+ NG2+ cells. To assess the relevance of NG2 at immune system and CNS levels, we induced EAE in bone-marrow chimeric mice, generated with WT recipients of NG2KO bone-marrow cells and vice versa. Regardless of their original phenotype, mice receiving NG2KO bone marrow developed milder EAE than those receiving WT bone marrow. Our data suggest that NG2 plays a role in EAE not only at CNS/BBB level, but also at immune response level, impacting on DC activation and thereby their stimulation of reactive T cells, through controlling IL-12 expression.

**Electronic supplementary material:**

The online version of this article (doi:10.1007/s00401-016-1563-z) contains supplementary material, which is available to authorized users.

## Introduction

The nerve/glial antigen 2 (NG2; also known as chondroitin sulfate proteoglycan 4, CSPG4) is a chondroitin sulfate proteoglycan expressed by a number of cell types within and outside the central nervous system (CNS). Within the CNS, NG2, together with platelet-derived growth factor receptor alpha (PDGFRα), is a marker of oligodendrocyte precursor cells (OPCs), which downregulate both markers as they differentiate into mature oligodendrocytes [16, 28, 39, 47]. The OPCs respond with increased proliferation to CNS injury and large numbers of NG2-expressing cells are found at sites of inflammation in the CNS [10]. In particular, OPC levels markedly increase at the acute phase of experimental autoimmune encephalomyelitis (EAE), the purported model for multiple sclerosis [16, 19], but are severely decreased in chronic EAE [16]. These cells have been shown to respond to demyelination by generating new oligodendrocytes [19, 27, 50, 55]. At the CNS microvessels, pericytes are involved in ensheathment of endothelial cells, blood–brain barrier (BBB)-endothelial cell differentiation, and maintenance of adult endothelial barrier function [2, 23]. Although NG2 is not expressed in adult CNS pericytes [53], it is an early marker of pericyte activation during CNS development and pathological conditions [40, 53]. Thus, upon CNS injury, NG2-reactive pericytes are found along microvessels, where they act as sensors for inflammation and support the immunosurveillance and effector function of extravasated neutrophils and macrophages [48]. Non-CNS resident infiltrating macrophages that express NG2 have also been observed in CNS inflammation [5, 33], but, to date, the expression of NG2 on other immune cell subsets has not been reported.

These observations suggest a role for NG2 in CNS injury associated with inflammation, a possibility corroborated by the accumulation of NG2 at the glial scar, due not only to the recruitment of OPCs, but also to the shedding of the extracellular domain of NG2 from OPCs and to its incorporation into the extracellular matrix [3, 9]. Moreover, there is controversial evidence for a role of NG2 in axon regeneration, remyelination, and CNS recovery [10]. Thus, in vitro studies have shown that NG2 inhibits neurite growth, suggesting that it could inhibit axonal regeneration [49, 52]. In contrast, other studies both in vitro [57] and in vivo [9, 24] suggest that NG2 is unlikely to be a major inhibitor of axonal regeneration after CNS injury.

On the basis of the above data, it appears highly likely that NG2 could play a role in EAE, a disease associated with increased BBB permeability, inflammatory infiltrates, and CNS damage. Moransard et al. studied EAE in NG2 knock-out (KO) mice to assess this possibility and did not observe any difference at clinical, immune response, and pathological levels, between NG2KO and wild-type mice [35]. In this study, we investigated EAE in NG2KO mice of the same origin [17] and found, in contrast, that NG2 plays an important role in EAE, with NG2 KO resulting in a significantly milder EAE disease, at clinical and neuropathological levels. Unexpectedly, we observed that NG2 significantly contributes to EAE pathogenesis and progression, not only through its expression on CNS-resident OPCs and pericytes, but also through its expression on immune cells, which had not been reported and studied previously, in particular at the encephalitogenic T cells/dendritic cells axis.

## Materials and methods

### Mice

Wild-type C57Bl/6J mice were purchased from Harlan (Bresso, Italy). The homozygous NG2^−/−^ mouse colony was generated as follows: NG2 knock-out (NG2KO) mice, developed by Stallcup and colleagues [17] on the C57BL/6J background and kindly provided by Prof. Malatesta (University of Genoa, Italy), were cross-bred with WT C57Bl/6J mice and the F1 heterozygous NG2^+/−^ mice were themselves crossed to obtain homozygous NG2^−/−^ F2 progeny. The NG2^−/−^ (NG2KO) mice were then interbred to maintain the homozygous colony and used for all experiments. NG2KO mice were regularly genotyped by PCR to ensure the maintenance of the insertion leading to gene disruption (Supplementary Fig. S1). The F2 NG2^+/+^ mice were themselves interbred to generate “wild-type” (WT) control mice. NG2KO mice were viable, healthy, fertile, and indistinguishable from WT mice. All mice were housed in pathogen-free conditions with food and water ad libitum. All applicable international, national, and/or institutional guidelines for the care and use of animals were followed (Decreto Legislativo 4 marzo 2014, n. 26, legislative transposition of Directive 2010/63/EU of the European Parliament and of the Council of 22 September 2010 on the protection of animals used for scientific purposes). The research protocol was approved by the Ethical Committee for Animal Experimentation of the University of Genoa, Italy.

### EAE induction

Chronic EAE was induced in female WT and NG2KO mice (6–8 weeks of age, weighing 18.5 ± 1.5 g) by subcutaneous injection at two different sites in the right and left flanks and one site at the tail base with an emulsion (300 µl total) containing 200 µg myelin oligodendrocyte glycoprotein peptide spanning amino acids 35–55 (MOG35–55) (Espikem) in incomplete Freund’s adjuvant (Sigma-Aldrich) supplemented with 300 µg *Mycobacterium tuberculosis* (strain H37RA; Difco). Mice were injected in the tail vein with 400 ng pertussis toxin (Sigma-Aldrich) in 100 µl of phosphate buffer saline solution (PBS, pH 7.6) immediately, and 48 h after the immunization. The mice were scored daily for clinical manifestations of EAE on a scale of 0–5 [59].

### Immunohistochemistry

Mice under deep anesthesia with ketamine/xylazine cocktail (90 mg and 4.5 mg/kg, respectively; intraperitoneal injection) were transcardially perfused with 100–150 ml of 2 % paraformaldehyde (PFA) and 0.2 % glutaraldehyde (Sigma-Aldrich) solution. Whole brains and spinal cords were removed and post-fixed by immersion in the same fixative at 4 °C for 4 h, then washed in PBS overnight at 4 °C, and the samples were stored in 0.2 % PFA in PBS at 4 °C. Spinal cord demyelination and inflammatory activity were evaluated as previously described [14, 59]. Briefly, demyelination was measured as the percent of demyelinated areas in the area of entire section of the spinal cord as follows: for each mouse (*n* = 5 mice per group), three sections per each of cervical, thoracic, and lumbar regions were stained with Luxol Fast Blue and demyelination was scored as follows: 1 = traces of subpial demyelination; 2 = marked subpial and perivascular demyelination; 3 = confluent perivascular or subpial demyelination; 4 = massive perivascular and subpial demyelination involving one half of the spinal cord with presence of cellular infiltrates in the CNS parenchyma; and 5 = extensive perivascular and subpial demyelination involving the whole cord section with presence of cellular infiltrates in the CNS parenchyma. In chimeric mice (see below), demyelination was assessed by staining for myelin basic protein (MBP) with anti-MBP antibody (diluted 1:90, Abcam). The inflammatory activity was analyzed by immunohistochemistry with anti–mouse CD3 (diluted 1:200, Santa Cruz) and anti–mouse IBA-1 (diluted 1:500, Abcam) antibodies, which identify T cells and microglia/macrophages, respectively. We used antibodies against glial fibrillary acidic protein (GFAP, diluted 1:500, Agilent Technologies) for the detection of astrocytes, and against inducible nitric oxide synthase (iNOS, diluted 1:100, Abcam) as inflammatory activity marker. All primary antibodies were detected with a biotin- or rhodamine-labeled secondary antibody (Bio-Rad) followed by diaminobenzidine-tetrahydrochloride detection according to standard protocol. Quantification was performed for the same areas as for demyelination in sequential sections of the same spinal cord regions, i.e., nine sections per animal, from approximately three segments apart. Images were captured at 40× magnification with an Axio Imager M1 microscope and analyzed with AxioCam software (Zeiss). The captured images were coded and quantified in a blinded manner. Data are expressed as mean ± standard error of the mean (SEM) of the total number of positive elements exclusively in the white matter and reported as number of positive elements per mm^2^.

To assess NG2 expression on immune cells, splenocytes were seeded onto poly-lysine-coated glass coverslips and then placed in 24-well plates in RPMI1640 (Thermo Fisher Scientific), 1 mM glutamine (Thermo Fisher Scientific), 2-mercaptoethanol (5 × 10^−5^ M, Sigma-Aldrich), penicillin/streptomycin (50 U/ml, Thermo Fisher Scientific), supplemented with 10 % fetal bovine serum (FBS, Thermo Fisher Scientific). After overnight incubation, cells were fixed with 4 % PFA and processed for immunocytofluorescence with rabbit anti-NG2 (diluted 1:150, Merck), anti-CD11b (diluted 1:100, Merck), anti-CD11c (diluted 1:200, Merck), and anti-CD3 (diluted 1:50, Santa Cruz) antibodies. Primary antibody binding was detected with the appropriate secondary antibody: IgG anti-rabbit FITC-conjugated for anti-NG2 and IgG anti-rabbit PE-conjugated for anti-CD11b, anti-CD11c, and anti-CD3 (diluted 1:100, Thermo Fisher Scientific). The reactivities were detected under an Axio Imager.M1 microscope and analyzed with AxioCam software (Zeiss).

#### Laser confocal microscopy immunofluorescence

These methods are essentially similar to what we previously described [11]. Briefly, the perfused brains and spinal cords were serially sectioned using a vibrating microtome (Leica) in about 120 serial sections (20 µm-thick) per sample and organized in a multiwell archive stored at 4 °C. Single and multiple immunostainings were carried out with the following primary antibodies: anti-CD45 (diluted 1:50; Novus Biologicals), anti-collagen type IV (Coll IV; diluted 1:50; Acris Antibodies GmbH), anti-PDGFRα (diluted 1:70; Millipore), anti-G-protein coupled receptor 17 (GPR17, diluted 1:350; Cayman Chemical), anti-Alzheimer precursor protein A4 (APP; diluted 1:100; Millipore), anti-MBP (diluted 1:90, Abcam), anti-CD13 (diluted 1:50; Aminopeptidase N; BD Pharmigen), anti-Claudin-5 (diluted 1:25; Invitrogen), anti-occludin (diluted 1:50; Invitrogen), anti-NG2 (diluted 1:200; Merck), and anti IBA-1 (diluted 1:50; Abcam) antibodies, then detected by appropriate fluorophore-conjugated (Alexa Fluor^®^ 488, 555 and 568; Invitrogen) or biotinylated (Vector Laboratories) secondary antibodies and fluorophore-conjugated streptavidin (Invitrogen) diluted in PBS containing 1 % bovine albumin and 2 % FBS. After immmunolabeling, the sections were counterstained with TO-PRO-3 (Alexa Fluor^®^ 633; diluted 1:10 in PBS; Invitrogen). Negative controls were prepared by omitting the primary antibodies, pre-adsorbing the primary antibodies with an excess of antigen when available, and mismatching the secondary antibodies. The sections were examined under a Leica TCS SP5 confocal laser-scanning microscope (Leica Microsystems) using a sequential scan procedure. Confocal images were taken at 250–500 nm intervals through the z-axis of the sections with 40× and 63× oil lenses.

#### Laser confocal microscopy morphometry

Quantitative assessment of PDGFRα^+^ OPCs, GPR17^+^ pre-myelinating oligodendrocytes, and MBP^+^APP^+^ axons was carried out through computer-aided morphometric analysis with Leica Confocal Multicolor Package (Leica Microsystems) and ImageJ software. Randomly chosen fields across the cerebral cortex and spinal cord white matter were analyzed for each mouse on 4 sections per brain and 3 sections per each of cervical, thoracic, and lumbar spinal cord regions. The number of positive cells and APP^+^ transversely cut axons was interactively counted (Cell counter ImageJ) on serial 0.5 μm optical sections across the entire stack of the projection images (20 μm thick). The results for the cells were normalized to the same volume (1 mm^3^), whereas the results for axons were normalized to a bi-dimensional value (reference area = 1 mm^2^).

### FITC-dextran injection

Mice were injected with a solution of heparin (100 Units/kg) containing 71-kDa FITC-labeled dextran (5 mg/ml, Sigma-Aldrich) into the tail vein. Two minutes later, the mice under deep anesthesia with ketamine/xylazine cocktail (90 mg and 4.5 mg/kg, respectively; intraperitoneal injection) were killed, and brain and spinal cord were collected immediately. The tissues were processed by post-fixation as described above, and 20-µm-thick slices were counterstained with TO-PRO3 (Alexa Fluor^®^ 633, diluted 1:10 in PBS; Invitrogen) and analyzed by confocal microscopy (Leica Microsystems) with 40× oil lens.

### T-cell proliferation assay

For the measurement of WT and NG2KO ex vivo proliferative responses, mice were immunized with MOG35-55 using the same protocol as for EAE induction, except that they did not receive pertussis toxin, and lymph node cells were isolated from the draining inguinal lymph nodes 9 days after immunization. MOG35–55-primed lymph node cells were cultured (2 × 10^5^ cells/well) in 96-well flat-bottom plates in the presence of 10 μg/ml MOG35–55, 1 μg/ml Concanavalin A (ConA, Sigma-Aldrich) as positive control, or 10 µg/ml proteolipid protein peptide spanning amino acids 139–151 (PLP), as negative control, for 3 days. Irradiated (25 Gy) splenocytes, isolated from the spleen of the same MOG-immunized mice and depleted of T cells through negative selection (Pan T cell Isolation Kit II, Miltenyi), were used as supplementary antigen-presenting cells (1 × 10^6^ cells/well). Cell proliferation was measured as DNA incorporation of 5-ethynyl-2′-deoxyuridine (EdU) with Click-iT^®^ EdU Microplate Assay (Thermo Fisher Scientific).

### Elisa

Cytokines were evaluated in supernatants from MOG35-55-primed WT and NG2KO T-cell cultures by ELISA Standard Set kits (IL-4, IL-17 and IL-10 ELISA MAX, Biolegend; IFN-γ, Mabtech). Briefly, 96-well ELISA plates coated overnight with appropriate capture antibodies at 4 °C were washed with PBS supplemented with 0.1 % Tween 20 and blocked with PBS containing 5 % FBS for 2 h at room temperature. Supernatants of cell cultures (100 μl) were added and the plates were incubated for 2 h at room temperature. Plates were washed and incubated with the relevant horseradish peroxidase-coupled detection antibodies for 1 h at room temperature. The plates were washed, substrate (3,3′,5,5′tetramethylbenzidine, Sigma-Aldrich) was added, and the plates were developed for 20–30 min. The reaction was stopped with 2 N H_2_SO_4_ and plates read at OD 450 nm on a Multilabel Victor3 reader (Perkin Elmer).

### Generation and activation of bone-marrow-derived dendritic cells

Primary bone-marrow-derived dendritic cells (BMDDCs) were obtained as described previously [6]. Briefly, at day 0, bone-marrow cells from WT and NG2KO mice were flushed from the femur and tibia and passed through a 70-µm nylon cell strainer (Becton–Dickinson, New Jersey, USA). The cell suspension was seeded in the presence of granulocyte macrophage colony-stimulating factor (20 ng/ml, Miltenyi) and after seven days, the cells were analyzed by FACS for surface marker CD11c. Purity of CD11c^+^ cells assessed by FACS with APC-conjugated anti-CD11c antibody (Becton–Dickinson) was at least 65 %. Activation of CD11c^+^ BMDDCs was then carried out by stimulation for 24 h with 10 μg/ml lipopolysaccharide (LPS, Sigma-Aldrich).

### Generation and polarization of bone-marrow-derived macrophages

To generate primary bone-marrow-derived macrophages (BMDMs), bone-marrow cells were flushed from the femur and tibia and passed through a 70-µm nylon cell strainer (Becton–Dickinson). The cell suspension was seeded in 6-well plates (6 × 10^6^/well) in the presence of macrophage colony-stimulating factor (100 ng/ml, Miltenyi) in Minimum Essential Medium alpha (Thermo Fisher Scientific) with 1 % penicillin/streptomycin (50 U/ml) and 10 % FBS. After 7 days, polarization of WT and NG2KO BMDMs towards M1 and M2 phenotypes was performed for 72 h by stimulation with IFN-γ (20 ng/ml, R&D Systems) or with IL-4 (20 ng/ml, R&D Systems), respectively. Polarization to the appropriate phenotype was confirmed by FACS analysis for the expression of M1- and M2-specific surface maturation markers (see below).

### FACS analysis

Surface marker staining and intracellular cytokine staining were performed using the Cytofix/Cytoperm, Fixation/Permeabilization Solution Kit (Becton–Dickinson), according to the manufacturer’s protocol. Briefly, cells (5 × 10^5^) were resuspended in 100 µl of FACS buffer (PBS, pH 7.2, containing 0.5 % bovine serum albumin) and stained with appropriate conjugated antibodies for 30 min at 4 °C.

To assess the NG2 expression on immune cells, splenocytes were isolated, passed through a 70-µm nylon cell strainer (Becton–Dickinson) to prepare a single-cell suspension, and used to optimize staining conditions. To establish background staining and to check the positive signal using the appropriate amount of anti-NG2 Alexa Fluor^®^488 antibody (diluted 1:100, Merck), we performed a limited set of pilot experiments where quadrants were set based on staining with isotype-matched control antibody and NG2KO splenocytes were tested as negative control. WT splenocytes were double-stained with anti-NG2 antibody and antibodies against relevant surface markers, PE-conjugated anti-CD3 (diluted 1:100, Biolegend), APC-conjugated anti-CD11c, and PE-conjugated anti-CD11b (diluted 1:100, Becton–Dickinson) antibodies, for T cells, DCs, and macrophages, respectively.

BMDMs from WT and NG2KO mice were assessed by FACS for surface-specific markers of macrophage maturation using PercP-conjugated anti-CD86, PE-conjugated anti-CD206 (diluted 1:100, both from Biolegend) and PE-conjugated anti-MHCII (diluted 1:100, Becton–Dickinson) antibodies. WT and NG2KO LPS-primed BMDDCs and MOG35–55-stimulated lymph node cells were incubated with APC-conjugated anti-CD11c antibody (diluted 1:100, Becton–Dickinson) for 30 min at 4 °C, fixed/permeabilized for 15 min at 4 °C, and processed for intracellular cytokine staining using PE-conjugated anti-IL-12 (p70) antibody (diluted 1:100, BD Pharmingen) for 30 min at 4 °C. Data were acquired on a FACS Canto II (Becton–Dickinson) and analyzed using DIVA 6.1 software.

### Real-time PCR

Total RNA was isolated using TRIzol^®^ Reagent (Thermo Fisher Scientific). First-strand cDNAs were generated starting from 1 μg RNA using Transcriptor First Strand cDNA Synthesis Kit (Roche). Real-time PCR was performed with 20 ng cDNA using a Roche Light Cycler 480 (Roche). Experiments were performed in triplicates, with glyceraldehyde 3-phosphate dehydrogenase (Gapdh) analyzed as housekeeping gene to normalize the expression data. The relative gene expression of target genes in reference to Gapdh expression was analyzed using the comparative CT threshold method [41], and the normalized expression was expressed as fold induction over the control sample. The following forward and reverse primers (TIB MOLBIOL) were used: Il12, forward primer: 5′-GGAGTCCAGTCCACCTCTACA-3′, reverse primer: 5′-ATCGTTTTGCTGGTGTCTCC-3′; Tnf, forward primer: 5′-GGTCTGGGCCATAGAACTGA-3′, reverse primer: 5′-TCTTCTCATTCCTGCTTGTGG-3′; Nos2, forward primer: 5′-TTCATGATAACGTTTCTGGCTCT-3′, reverse primer: 5′-TGAACTTGAGCGAGGAGCA-3′; Chil3, forward primer: 5′-GAGACCATGGCACTGAACG-3′, reverse primer: 5′-AAGAACACTGAGCTAAAAACTCTCCT-3′; Arg1, forward primer: 5′-GAATCCTGGTACATCTGGGAAC-3′, reverse primer: 5′-GAATCTGCATGGGCAACC-3′; Ifng, forward primer: 5′-TTCAAGACTTCAAAGAGTCTGAGG-3′, reverse primer: 5′-GGAGGAACTGGCAAAAGGAT-3′; Il1b, forward primer: 5′-TTTGAAGCTGGATGCTCTCAT-3′, reverse primer: 5′-AGTTGACGGACCCCAAAAG-3′; Sox, forward primer: 5′-AGCCTCATCGGAGGGCTA-3′, reverse primer: 5′-AGGCAACTGCAGGCTGTAAAA-3′; Gapdh: forward primer: 5′-AATCTCCACTTTGCCACTGC-3′, reverse primer: 5′-ATGGTGAAGGTCGGTGTGA-3′.

### Generation of chimeric mice

To create bone marrow chimera, female WT and NG2KO recipient mice (8 weeks of age) were irradiated with a lethal dose (9.5 Gy) and immediately transplanted with bone-marrow cells (5 × 10^6^ per mouse in 100 µl of PBS injected in the tail vein) isolated from hind legs of syngeneic 8-week-old donor male mice. Reconstitution in the recipient was evaluated 8 weeks later by real-time PCR for sex-determining region Y (Sox) gene [37] in circulating leucocytes. To avoid host infections, 50 mg/ml Gentamicin (Sigma-Aldrich) was added to the drinking water, 1 week before irradiation and for 2 weeks thereafter.

### Statistical analysis

The results were analyzed with GraphPad Prism software (version 6.01; GraphPad Software). Intergroup differences were analyzed by one-way ANOVA followed by Bonferroni’s post hoc test. Comparison of two sets of data used the Mann–Whitney test. Differences between groups were considered to be significant when *P* ≤ 0.05 (*), *P* ≤ 0.01 (#) and *P* ≤ 0.0001 (**).

## Results

### NG2KO mice develop milder EAE than their WT counterparts

**Fig. 1:**
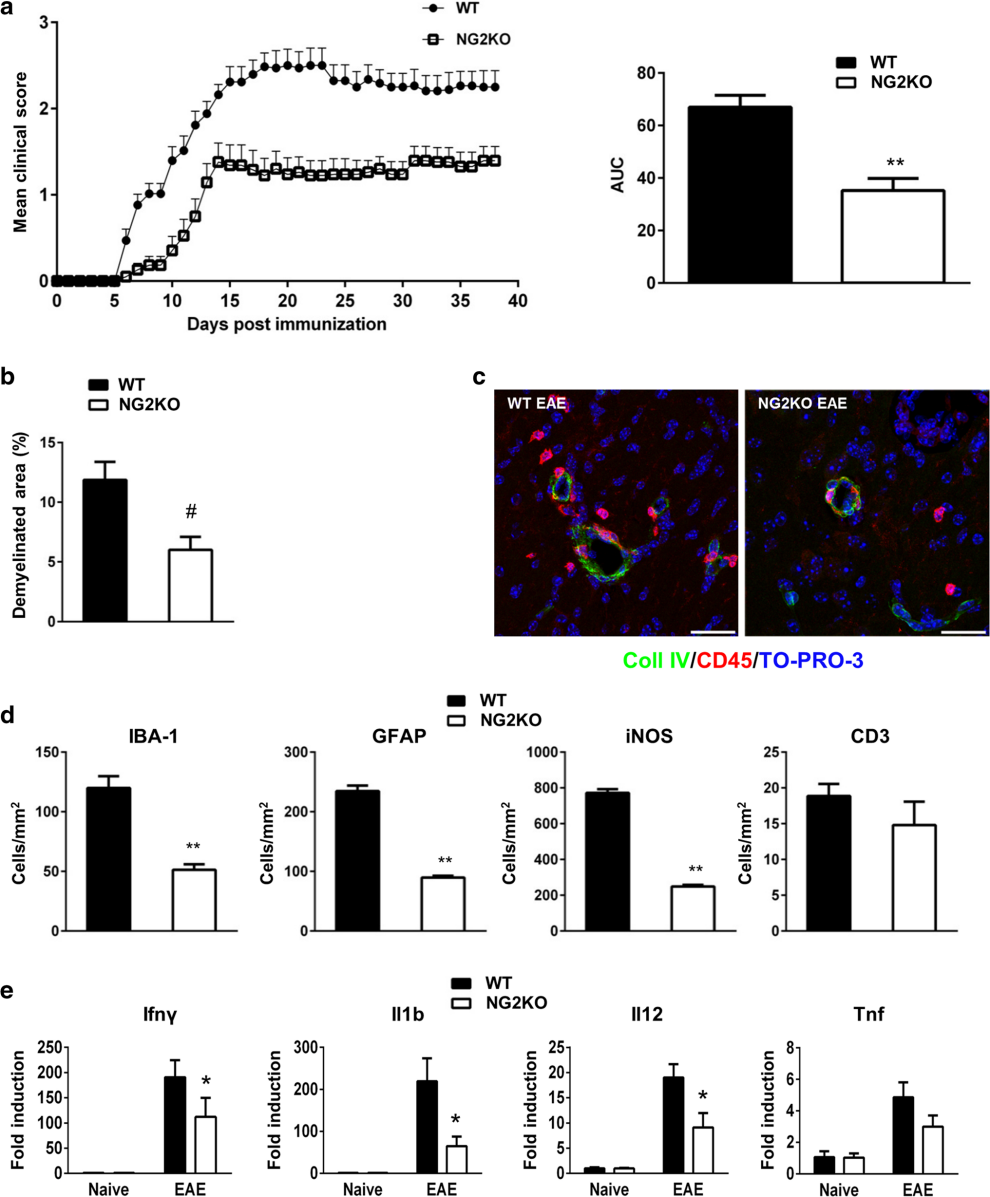
NG2KO mice develop milder EAE than their WT counterparts. **a** MOG-induced EAE follows a less severe course in NG2KO mice. A representative of three independent experiments is presented (WT, *n* = 17; NG2KO, *n* = 19. Total mice tested in three experiments: WT, *n* = 33; NG2KO, *n* = 35). Data are shown as mean ± SEM daily clinical score (*left panel*) and mean ± SEM area under the curve (AUC) of EAE clinical course calculated for each mouse (*right panel*). **P < 0.0001. **b** Demyelination is decreased in NG2KO mouse CNS. Demyelinated areas were quantified by Luxol Fast Blue staining for myelin in spinal cord sections from WT and NG2KO EAE-affected mice (3 sections per each of cervical, thoracic and lumbar regions per mouse, *n* = 5 mice per group) sampled at chronic disease phase, 40 days post EAE induction. Data are expressed as mean ± SEM percent of demyelination (weakly stained area over strongly stained area in all spinal cord sections assessed × 100). ^#^ *P* < 0.01. **c** Representative confocal microscopy images showing perivascular infiltration of CD45^+^ cells in WT and NG2KO brain. In EAE-affected WT mice (40 days post induction), an inflammatory infiltrate is evidenced around microvessel stained for collagen IV (Coll IV) as a component of the basement membrane, whereas in EAE-affected NG2KO mice, perivascular CD45-positve cells are rarely observed (Coll IV, *green*; CD45, *red*). *Scale bar* 20 μm. **d** Quantification of confocal microscopy data shows a decrease in inflammatory cells, but not infiltrating T cells in NG2KO mouse CNS. Spinal cord sections as in **b** were stained for the presence of microglia/macrophages (IBA-1), activated astrocytes (GFAP), stress marker-positive cells (iNOS), and infiltrating T-cells (CD3). Data are presented as mean ± SEM positive cell number/mm^2^. **P < 0.0001. **e** In EAE, the NG2KO phenotype is associated with decreased expression levels of pro-inflammatory cytokines in the CNS. Real-time PCR analysis of mRNA expression of genes associated with inflammation in EAE spinal cord. mRNA was obtained from whole spinal cord samples collected at early-disease phase, 6 days after disease onset. Data are presented as mean ± SEM fold induction over naïve WT value of 3 independent experiments. *P < 0.05

Figure 1a (left panel) shows a representative clinical course of EAE in WT and NG2KO mice. While both groups exhibited a chronic course of EAE, there were major differences in incidence, onset, and clinical severity. In the three experiments performed (total *n* = 33 WT and 35 NG2KO), disease incidence was lower in NG2KO mice (84 ± 4.5 vs. 100 % in WT mice) and disease onset was delayed (13.06 ± 3.66 vs. 8.16 ± 1.71 days for WT mice). Most importantly, the disease followed a significantly less severe course in NG2KO mice than in WT mice throughout the 40-day monitoring period, as assessed by measurement of the area under the curve (AUC; Fig. 1a, right panel). Histological examination of CNS tissue at the chronic stage of the disease (day 40 post-immunization) showed significantly reduced demyelination in the spinal cord of NG2KO mice (Fig. 1b), which was accompanied by lower numbers of inflammatory cells in the CNS (Fig. 1c, d and Supplementary Fig. S2). Thus, a decrease in perivascular extravasation of leukocytes (CD45^+^ cells), possibly macrophages, in NG2KO mouse brain was observed by confocal microscopy (Fig. 1c). Similarly, the numbers of IBA-1^+^ and iNOS^+^ cells, most likely microglia, were reduced by two- to fourfold in the spinal cord of NG2KO mice (Fig. 1d and Supplementary Fig. S2) and GFAP^+^ reactive astrocytes were significantly decreased in EAE-affected NG2KO mice compared to WT controls (Fig. 1d). At this chronic stage of the disease, there was no difference in the number of infiltrated T cells (CD3^+^) in CNS of EAE-affected NG2KO as compared to WT mice (Fig. 1d). These results, therefore, show that actively induced EAE is considerably attenuated in NG2-deficient mice, both at clinical and neuropathological levels, and contrast with those of a previous study in which NG2 was found to be dispensable for EAE [35].

### EAE-associated neuroinflammation is less intense in spinal cord of NG2KO mice

We sought to evaluate if the decreased numbers of inflammatory cells in EAE-affected NG2KO CNS reflected on the inflammatory milieu of EAE-affected WT vs NG2KO CNS by measuring mRNA expression of EAE-relevant pro-inflammatory cytokines, Ifng, Tnf, Il1b, and Il12. The expression of all four genes did not differ in naïve WT vs NG2KO spinal cord and greatly increased in both WT and NG2KO at the early phase of EAE as expected (Fig. 1e). However, the expression of the four genes in EAE was lower in NG2KO spinal cord than that in WT spinal cord (Fig. 1e), with significant differences observed for Ifng, Il1b, and Il12, confirming that the CNS environment in EAE-affected NG2KO mice is characterized by reduced inflammation, as compared to that of EAE-affected WT mice.

### OPC numbers do not change in NG2KO brain during EAE

**Fig. 2:**
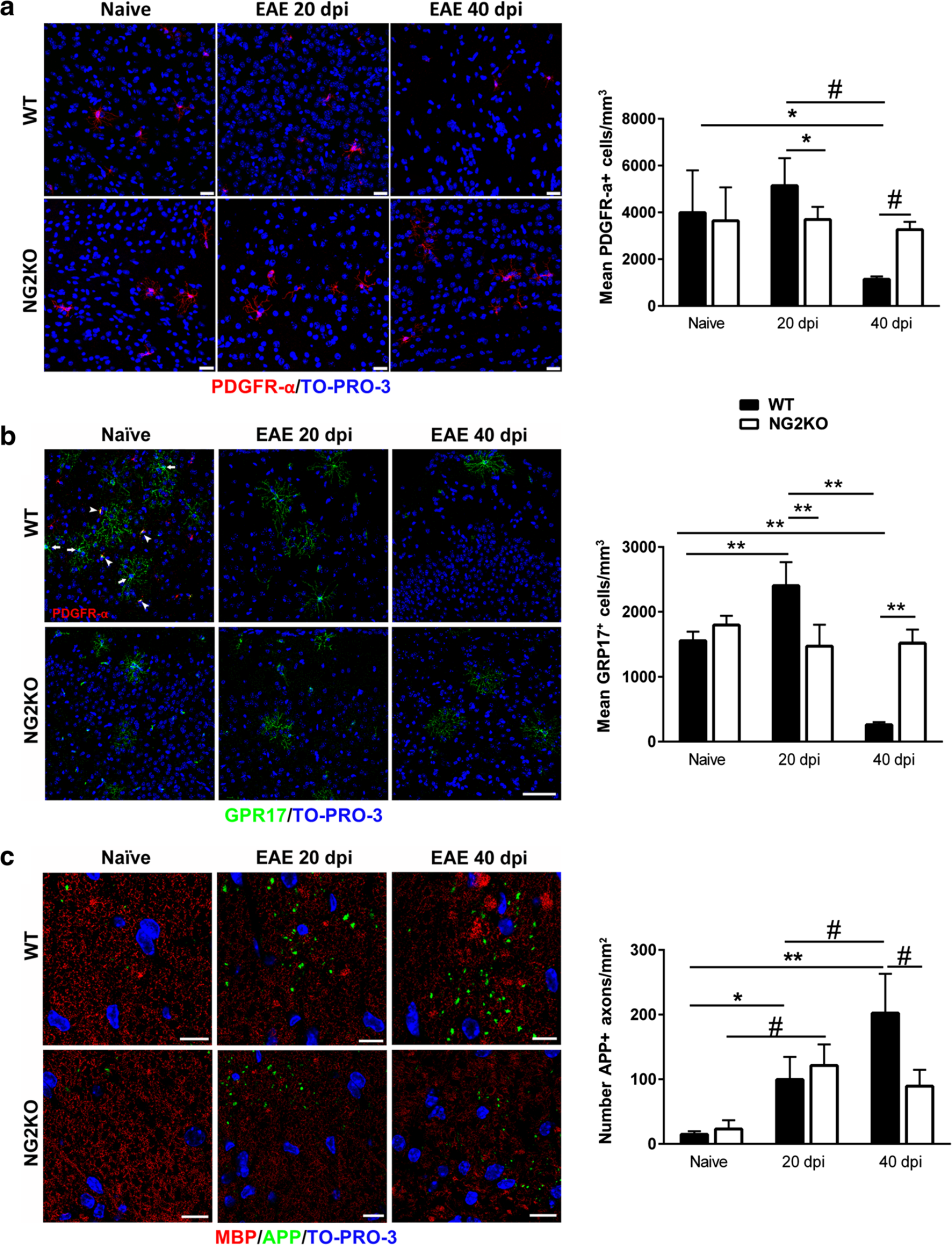
Decrease in OPCs at EAE chronic stage does not occur in NG2KO mice. **a** OPCs quantified by confocal microscopy morphometry on brain PDGFRα-stained sections **(** *left panel* **)** show a sharp decrease in EAE-affected WT mice at the chronic disease stage, while their number remains constant in EAE-affected NG2KO mice throughout the disease (*right panel*). OPCs were quantified in cerebral cortex of naïve and EAE mice (4 sections per mouse, *n* = 5 mice per group) by counting PDGFRα^+^ cells, as justified by confocal microscopy data that confirmed the co-localization of NG2 and PDGFRα in WT mouse brain (Supplementary Fig. S3a). Similar results were obtained from the quantification of OPCs in spinal cord (Supplementary Figure S3c). *Scale bars* 25 μm. Data are presented as mean ± SD cell number/mm^3^. *P < 0.05, ^*#*^ *P* < 0.01. **b** Numbers of pre-myelinating oligodendrocytes do not change in NG2KO mice throughout EAE. Pre-myelinating oligodendrocytes were quantified in cerebral cortex of naïve and EAE mice (4 sections per mouse, *n* = 4 mice per group) by staining for GPR17 and counting only highly ramified GPR17^+^ cells. This is justified through confocal microscopy of naïve WT tissue stained concomitantly for PDGFRα, which shows that pre-myelinating highly ramified cells with broad GPR17 expression (*left panel*, *arrows*) do not express PDGFRα, whereas GPR17 expression in PDGFRα^+^ OPCs appears as single intracellular spots (*left panel*, *arrow heads*) [4]. Data (*right panel*) are presented as mean ± SD cell number/mm3. **P < 0.0001*. Scale bar* 75 μm. **c** The extent of axonal damage at EAE chronic phase is reduced in NG2KO CNS. Nerve fibers were localized by MBP reactivity and damaged axons were quantified as APP^+^ transversely cut axons (*left panel*) in spinal cord white matter of naïve and EAE mice (4 sections per mouse, *n* = 4 mice per group). Data (*right panel*) are presented as mean ± SD axon number/mm^2^. *P < 0.05, ^*#*^ *P* < 0.01, **P < 0.0001. *Scale bars* 10 μm. All quantifications were performed at both early (20 dpi) and chronic (40 dpi) disease phases

We have previously shown that in WT mice, the progressive process of demyelination was accompanied by a strong reduction in the number of OPCs at the chronic EAE phase (39 days post-induction) after a significant increase at the early EAE phase [16]. To analyze whether the lack of NG2 affected this important response of the oligodendrocyte cell lineage, we quantified the number of OPCs in both WT and NG2KO mice by immunofluorescence confocal microscopy. We first verified the concomitant expression of NG2 and an alternative OPC marker, platelet-derived growth factor receptor-α (PDGFRα), in WT OPCs [28] to validate the subsequent quantification of OPCs in NG2KO mice (Supplementary Fig. S3a, left panel). We assessed the lack of NG2 expression in NG2KO OPCs by NG2/PDGFRα double-labeling (Supplementary Fig. S3a, right panel), as well as in NG2KO brain microvessels by double-staining for NG2 and the pericyte marker CD13 (Supplementary Fig. S3b). As expected from our PCR analysis, which confirmed the gene disruption in our mice (Supplementary Fig. S1), no NG2-specific signal was detected in the CNS of NG2KO mice, either in OPCs or in pericytes (Supplementary Fig. S3a and b). Quantification of cell numbers, based on confocal microscopy computer-aided morphometry (Fig. 2a, left panel), showed no significant differences in PDGFRα^+^ OPCs in naïve WT and NG2KO mice. During EAE, the number of OPCs in brain and spinal cord showed an increase at 20 dpi and a significant decrease at 40 dpi in WT mice, as expected [16]. In contrast, OPC numbers remained constant in NG2KO mice throughout the disease (Fig. 2a right panel and Supplementary Fig. S3c). These results suggest that the lack of NG2 on OPCs may not only affect their proliferative ability and/or their differentiation to pre-myelinating oligodendrocytes, but also may reduce OPC apoptosis during the chronic phase of EAE, possibly due to a less inflammatory CNS milieu in these mice. Evaluation of GPR17^+^ pre-myelinating oligodendrocytes (Fig. 2b) indicated a parallel with our observations for OPCs. Thus, while in EAE-affected WT mice GPR17^+^ pre-myelinating oligodendrocytes increased at 20 dpi and decreased sharply at 40 dpi, their numbers did not change in NG2KO EAE-affected mice (Fig. 2b, right panel), suggesting that the lack of NG2 does not affect the differentiation ability of NG2KO OPCs. A reduced loss of pre-myelinating oligodendrocytes together with reduced inflammation and demyelination might suggest that axonal loss could also be lower in NG2KO mice. Indeed, we observed that, while axonal damage increased in WT EAE-affected mice throughout the disease, the number of damaged axons was markedly lower in NG2KO mice at the chronic phase (Fig. 2c).

### The preserved continuity of tight-junction protein distribution in EAE NG2KO CNS is associated with an efficient blood–brain barrier function

**Fig. 3:**
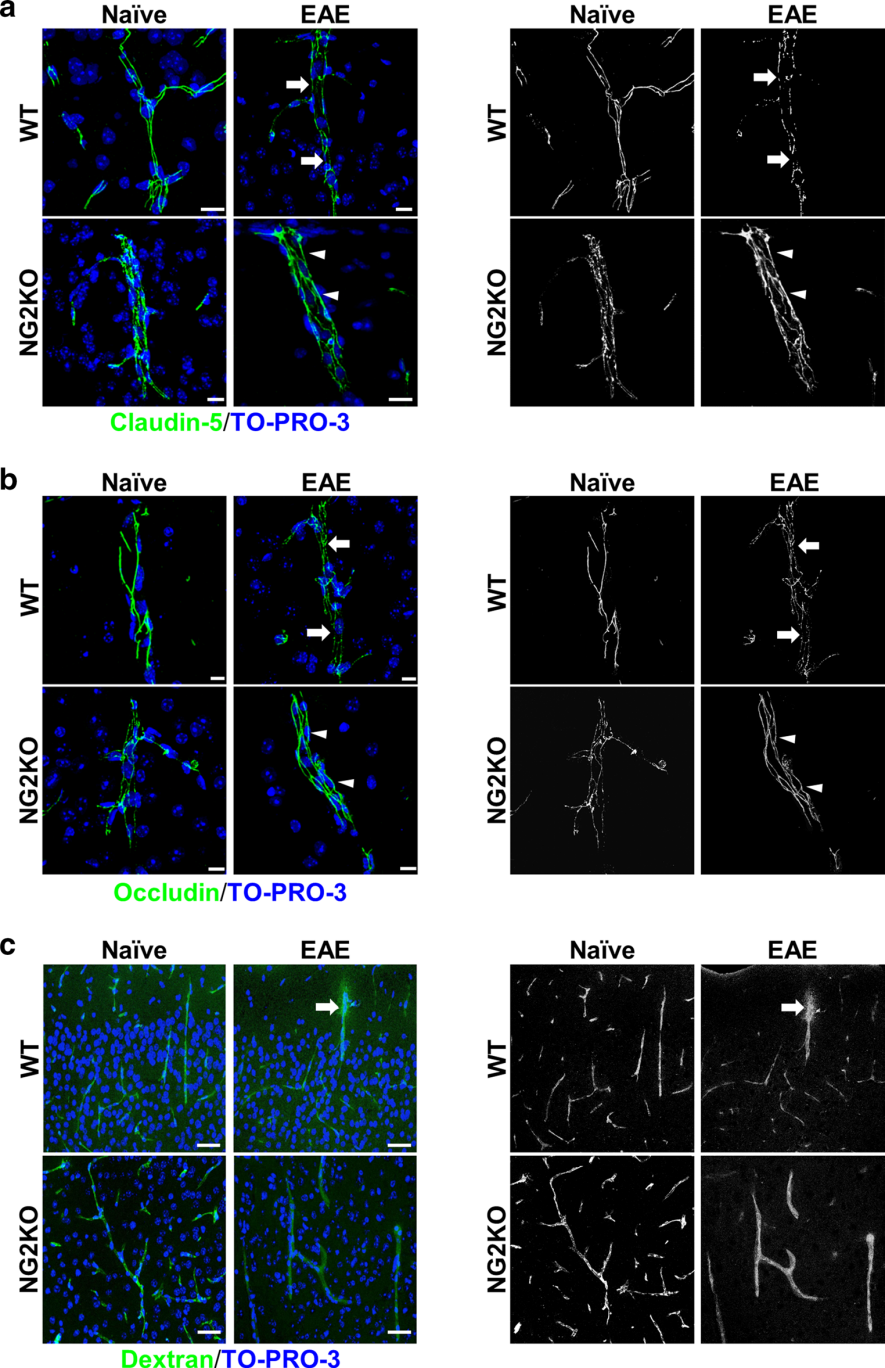
The preserved continuity of tight-junction protein distribution in CNS of EAE-affected NG2KO mice is associated with an efficient blood–brain barrier function. **a**, **b** Confocal microscopy images of cerebral cortex immunostained for claudin-5 and occludin in naïve and EAE-affected WT and NG2KO mice at chronic disease stage (40 dpi). In naïve WT mice, staining for claudin-5 and occludin shows a continuous, linear, and regular pattern, whereas in EAE-affected WT mice the staining pattern is discontinuous, characterized by thin tracts and unlabeled gaps (*arrows*). In naïve NG2KO mice, claudin-5 and occludin reactivity appears continuous although irregular, with thin fluorescent tracts alternating with points of focal staining; in EAE-affected NG2KO mice, the junctional immunostaining is continuous and regular (*arrowheads*). **c** Confocal microscopy images of cerebral cortex of mice injected with FITC-dextran confirms the preserved BBB function in EAE-affected NG2KO mice. FITC-dextran leakage in EAE-affected WT mice (*arrow*) is not observed in EAE-affected NG2KO mouse brains. Nuclei are stained with TO-PRO-3 (*blue*). *Scale bars* **a** 20 μm; **b** 10 μm; and **c** 30 μm. The binary image rendering shown in the right panels emphasizes the changes in expression of tight-junction proteins among all the experimental groups and the discontinuity of the EAE-affected WT pattern vs the EAE-affected NG2KO pattern, as well as the diffuse leakage in EAE-affected WT mouse brain

Because NG2^+^ pericytes play an important role in the formation of the neurovascular unit and BBB, we sought to evaluate if the reduced level of neuroinflammation in EAE-affected NG2KO mice could be related to specific alterations in the BBB. Thus, we investigated the distribution of the major structural and functional proteins of endothelial tight-junction (TJ), claudin-5 and occludin [12], in cerebral cortex and spinal cord microvessels of WT and NG2KO mice by immunofluorescence confocal microscopy. On confocal projection images of naïve WT BBB microvessels, the fundamental arrangement of claudin-5/occludin at the junctional membranes formed a ribbon-like regular and continuous staining pattern, which was lost in EAE-affected WT mice where it changed to a discontinuous one, characterized by claudin-5/occludin-reactive short tracts and punctate rows ([11] and Fig. 3a, b). In naïve NG2KO mice, the staining for claudin-5 and occludin, while continuous, was characterized by thin lines and focal spots (Fig. 3a, b and Supplementary Fig. S4). In EAE-affected NG2KO mice compared with EAE-affected WT mice, claudin-5 and occludin staining patterns were preserved and in some microvessels appeared even more regular than in naïve NG2KO mice, being formed by claudin-5/occludin-reactive wide junctional strips along the entire length of the microvessels examined (Fig. 3a, b and Supplementary Fig. S4). In agreement with the described continuous staining pattern, purported to represent the sealing function of TJ strands, a preserved barrier function, as demonstrated by Dextran-FITC experiments, was observed in naïve and EAE-affected NG2KO mice (Fig. 3c). Indeed, disruption of BBB-endothelial cell TJs and leakage of the permeability tracer described in cerebral cortex and spinal cord microvessels of EAE-affected WT mice ([11] and Fig. 3c, upper right panel) was never seen in naïve or EAE-affected NG2KO mice, the fluorescent Dextran-FITC signal being always restricted to the vessel lumen (Fig. 3c, lower left and right panel, respectively). Overall, these results emphasize the barrier efficiency to the exogenous tracer of the continuous distribution of TJ proteins in naïve and EAE-affected NG2KO mice. We suggest that in NG2KO mice, the lack of NG2 on pericytes affects the dynamic assembly of TJs, and upon pericyte activation during EAE, alternative signaling transduction pathways involved in TJ maintenance are triggered that result in the targeting of TJ molecules to the TJ.

### NG2 is expressed by immune cells

**Fig. 4:**
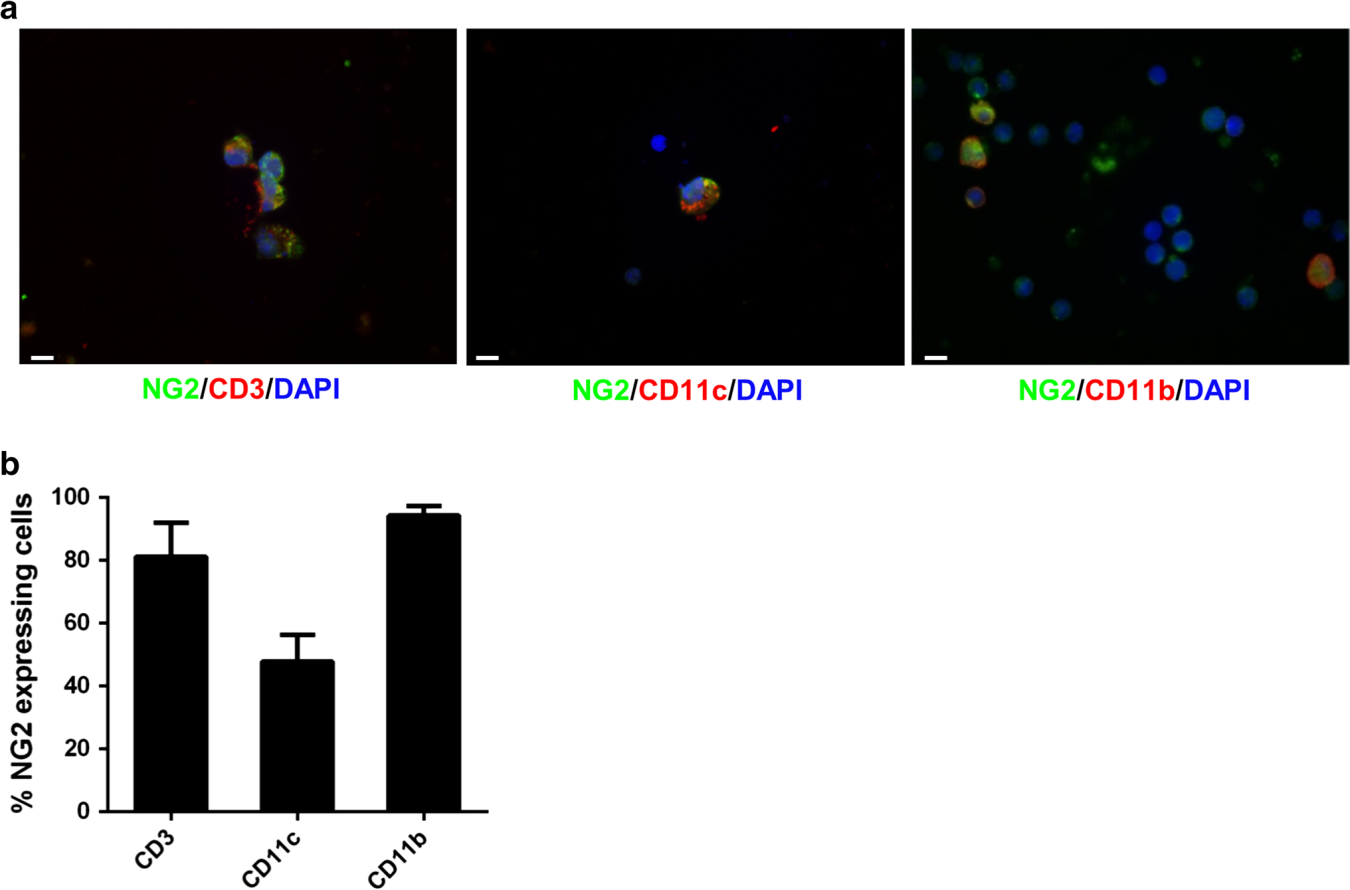
NG2 is expressed by immune cells. **a** Confocal microscopy images demonstrate the expression of NG2 (*green*) in T cells (CD3, *red*, *left panel*), dendritic cells (CD11c, *red*, *middle panel*), and macrophages (CD11b, red, right panel) within WT splenocytes. *Scale bar* 5 μm. Magnification ×100. Confirmation of the lack of NG2 expression on NG2KO immune cells, and thereby the specificity of the anti-NG2 antibody, is presented in Supplementary Fig. S5. **b** FACS analysis confirms that the majority of mouse T cells and macrophages, as well as a large proportion of dendritic cells, express NG2. Quantitative histogram of NG2 expression on WT T cells (CD3) freshly isolated from spleen, and in vitro-differentiated BMDDCs (CD11c) and BMDMs (CD11b). Data are presented as mean ± SEM of four independent experiments

In the injured CNS, in addition to OPCs and pericytes, NG2 is expressed by invading macrophage-like cells [5, 28, 33]. The possibility was raised that NG2 null macrophages may exhibit less inflammatory properties than their WT counterparts [28], leading to a reduced ability to invade the CNS [30], and such a feature would support the lower extent of inflammation in NG2KO CNS during EAE. To determine if the possible role of NG2 in inflammation might also be relevant for other inflammatory cells that are liable to infiltrate the CNS during EAE [7, 56], we sought to ascertain whether NG2 is expressed in WT mice or not by cells of the systemic immune system. We assessed the expression of NG2 by T cells in freshly isolated splenocytes and by bone-marrow-derived dendritic cells (BMDDCs) and macrophages (BMDMs). As can be seen in Fig. 4, confocal microscopy analysis with antibodies against markers of T cells (CD3), BMDDCs (CD11c), and BMDMs (CD11b) showed that each of these cell types expressed NG2 (Fig. 4a), and quantification by FACS revealed that the majority of T cells and BMDMs were NG2^+^, whereas around 50 % of BMDDCs expressed it (Fig. 4b). Similar data were obtained for CD11c^+^ and CD11b^+^ cells in freshly isolated splenocytes (data not shown). These data suggest that the decreased EAE pathology in NG2KO mice might not only be caused by differences at the CNS and BBB levels, but could also be related to the lack of NG2 expression on disease-relevant immune cells.

### The encephalitogenic T-cell response to MOG in NG2KO mice is shifted towards a less inflammatory profile

**Fig. 5:**
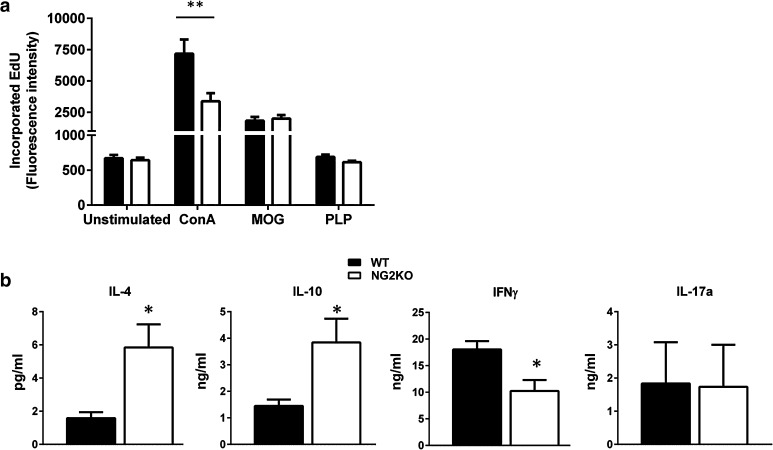
Lack of NG2 on T cells does not affect their antigen-specific proliferation but affects their cytokine profile. **a** Ex vivo analysis of the recall response by lymph node cells isolated from MOG35-55-primed WT and NG2KO mice 9 days post-immunization. Data of proliferation to MOG35-55 (MOG, 10 μg/ml) are presented as mean ± SEM incorporated EdU of three independent experiments. Response to concanavalin A (ConA, 5 μg/ml) and proteolipid protein peptide 139–151 (PLP, 10 μg/ml) are presented as positive and negative controls for the assay, respectively. **P < 0.001. **b** The cytokine profile of NG2KO lymph node cells in response to MOG is shifted towards a non-inflammatory type as compared to WT cells. Supernatants from the 3-day proliferation assays in (**a**) were assessed by ELISA for their cytokine contents. A representative of the three independent experiments is shown. Data are presented as mean ± SEM cytokine concentration of quadruplicate supernatant samples. *P < 0.05

To determine if the lack of NG2 on immune cells could impact disease expression in NG2KO mice, we compared T-cell proliferation and cytokine expression in response to the encephalitogenic peptide. Lymph node cells, isolated from WT and NG2KO mice primed 9 days previously with MOG35–55, were assessed for their ex vivo recall proliferation to MOG35-55 and to positive (mitogenic stimulation with ConA) and negative (stimulation with PLP139-151, another myelin antigen) T-cell stimuli. Cells isolated from WT and NG2KO mice proliferated equally well to MOG35–55 (Fig. 5a), suggesting that the antigen-specific proliferative response is not dependent on NG2 expression on T cells. The mitogenic response to ConA by WT cells was generally higher than that by NG2KO cells, and we can only speculate that this might be related to increased activation of WT T cells upon binding by ConA not only to the CD3, but also to NG2 [22, 34, 36]. In contrast, the cytokine profile of MOG35-55 reactive WT and NG2KO T cells differed significantly. As can be seen in Fig. 5b, the concentration measured by ELISA of the pro-inflammatory Th1-like cytokine, IFN-γ, was significantly lower in supernatants of the 3-day ex vivo recall response to MOG35–55 by NG2KO T cells, whereas those of the anti-inflammatory, Th2-type cytokines, IL-4 and IL-10, were significantly elevated, as compared to WT cells. IL-17a levels did not differ.

These findings suggest that in NG2KO mice, the T-cell response to the encephalitogenic MOG35-55 is skewed towards a Th2-cell response, which is notoriously associated with reduced encephalitogenicity and/or contributes to disease recovery.

### IL-12 expression is reduced in stimulated NG2KO DCs. Role of NG2 in DC activation

**Fig. 6:**
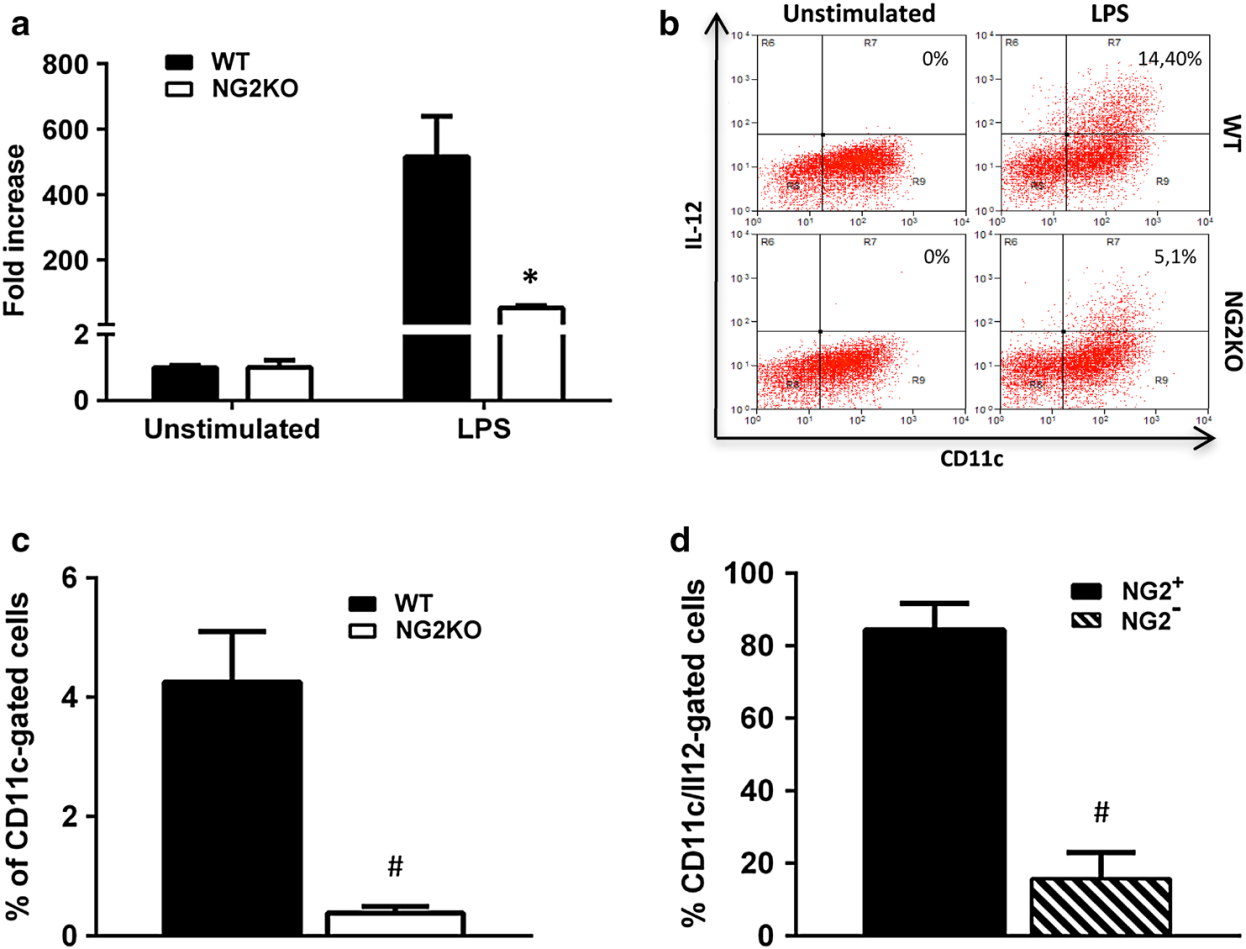
The expression of IL-12 is reduced in NG2KO DCs. **a** Real-time PCR and **b** intracellular FACS analyses show reduced expression of IL-12 in LPS-stimulated BMDDCs from naïve NG2KO mice. BMDDCs seeded in 6-well plates (7.5 × 10^6^ cells/well) were stimulated with LPS (10 µg/ml) for 24 h. Real-time PCR data are presented as mean ± SEM fold increase over unstimulated cells of three independent experiments; *P < 0.05. FACS plots of a representative of three independent experiments are presented. **c** Lack of NG2 on DCs isolated from lymph nodes in MOG35–55-primed NG2KO mice leads to their reduced expression of IL-12. Lymph node cells isolated from WT and NG2KO mice primed with MOG35–55 7 days before were analyzed by FACS for concomitant expression of surface CD11c and intracellular IL-12. Quantitative data are presented as mean ± SEM % of IL-12-expressing CD11c^+^ cells of three independent experiments. **d** In WT mice, the proportion of IL-12-expressing cells is lower in CD11c^+^ cells that do not also express NG2. MOG35–55-primed WT lymph node cells as in (**c**) were stained concomitantly for intracellular IL-12, NG2, and CD11c and analyzed by FACS. The CD11c^+^ cells within the IL-12-gated population were analyzed for their NG2 expression. Quantitative data are presented as mean ± SEM % of IL-12-gated cells that expressed CD11c and were NG2^+^ or NG2^−^, for 3 independent experiments

The cytokine shift of encephalitogenic NG2KO T cells is unlikely to be a feature intrinsic to these T cells themselves, as there was no difference in cytokine profile of MOG35–55-specific T-cell lines generated from WT and NG2KO mice (Supplementary Fig. S6). To assess whether it could instead be related to their stimulation upon antigen presentation, we investigated the expression of IL-12, the cytokine involved in the differentiation of naïve T cells into Th1 cells, by activated NG2KO DCs as compared to their WT counterparts. BMDDCs were stimulated or not with LPS and IL-12 expression was assessed by real-time PCR and intracellular FACS analyses (Fig. 6a, b). There was a significant decrease in IL-12 mRNA expression by three to fourfolds in NG2KO BMDDCs (Fig. 6a) that was corroborated by a parallel decrease at the protein level (Fig. 6b), indicating that NG2KO DCs are less capable of inducing a Th1 response than WT DCs. To assess if this in vitro feature of NG2KO DCs reflected the in vivo situation, we immunized WT and NG2KO mice for EAE, isolated CD11c^+^ cells from the draining lymph nodes 7 days later, and analyzed their IL-12 expression by intracellular FACS. As can be seen in Fig. 6c, the proportion of IL-12-expressing CD11c^+^ cells was significantly decreased in MOG35-55-primed NG2KO lymph node cells as compared to WT. Whether this might be due to an inherent lower expression of the cytokine or to a reduced migration of the DCs to the lymph nodes in NG2KO mice is unclear at this stage, but suggests that these cells might be less effective to induce in vivo differentiation to Th1 cells. Based on these data and our observation that 50 % of CD11c^+^ cells express NG2 (Fig. 4b), we wondered if the expression of IL-12 by DCs might be related to their expression of NG2. Accordingly, we quantified the percent of NG2^+^ and NG2^−^ CD11c^+^ cells in the IL-12-gated population of lymph node cells isolated from MOG35-55-primed WT mice. Interestingly, the proportion of IL-12-expressing cells was very low in CD11c^+^ cells that did not express NG2 and significantly lower than that in NG2^+^ CD11c^+^ cells (Fig. 6d). Altogether, these data suggest that NG2 expression associates with the ability of DC to produce sufficient amounts of IL-12 to properly prime encephalitogenic Th1 cells.

### NG2 is not involved in macrophage polarization

**Fig. 7:**
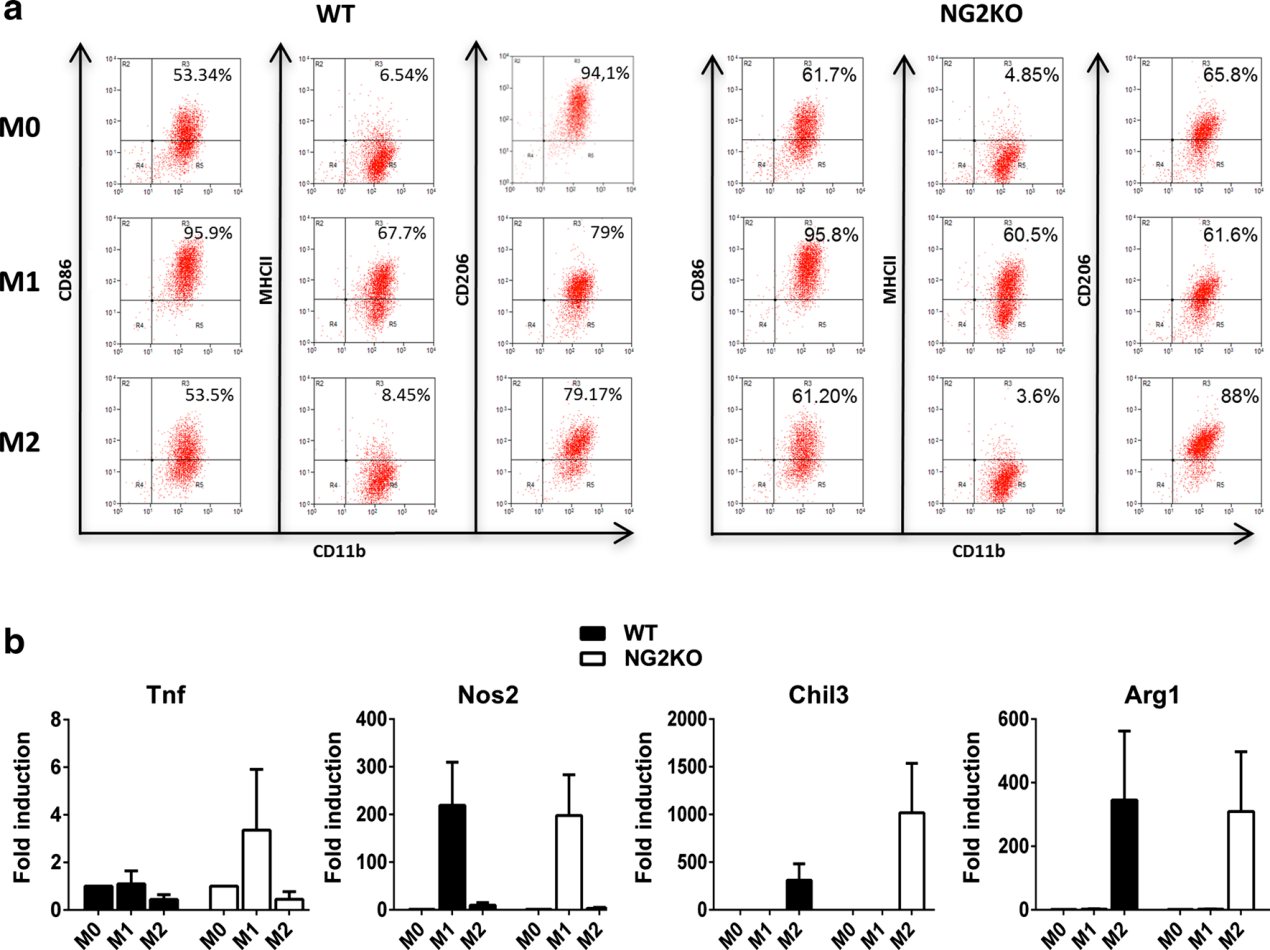
Lack of NG2 does not affect macrophage polarization in vitro. **a**, **b** FACS and real-time PCR analyses show that the expression of M1 and M2 markers does not differ between WT and NG2KO polarized BMDMs. WT and NG2KO BMDMs seeded in 6-well plates (6.0 × 10^6^ cells/well) were stimulated or not with IFN-γ (M1) or IL-4 (M2) (both at 20 ng/ml) for 48 h. **a** FACS plots, representative of three independent experiments, of naïve (M0) and M1- and M2-polarized BMDMs stained for M1 (CD86, MHCII) and M2 (CD206) surface markers; **b** mRNA expression of M1 (Tnf, Nos2) and M2 (Chil3, Arg1) markers in polarized BMDMs. Data are presented as mean ± SEM fold induction over M0 value, of three independent experiments

Our demonstration that the number of cells positive for IBA-1, marker for activated microglia/macrophages, was significantly lower in CNS of EAE-affected NG2KO mice (Fig. 1d) suggested the possibility that, in addition to decreased microglia activation, the number of infiltrating activated macrophages was lower in these mice during EAE, as also supported by a decreased number of perivascular CD45^+^ cells (Fig. 1c). While this observation could be secondary to the integrity of the BBB we observed in NG2KO mice, and to a decreased interaction of invading macrophages with endothelial adhesion molecules that are needed for extravasation [30], it could also be related to a defect in macrophage activation and polarization. Such a possibility would also impact on antigen-presenting function of NG2KO macrophages and, therefore, on the encephalitogenicity of the T-cell response. Accordingly, we sought to determine whether NG2 plays a role or not in macrophage polarization. BMDMs were polarized towards the M1 or M2 phenotype by priming with IFN-γ or IL-4, respectively, and assessed for the expression of M1 and M2 markers. As can be seen in Fig. 7a, FACS analysis for M1 (CD86, MHCII) and M2 (CD206) surface markers did not reveal any difference in unprimed WT vs NG2KO BMDMs (M0), IFN-γ-primed WT vs NG2KO BMDMs (M1), or IL-4-primed WT vs NG2KO BMDMs (M2). Similarly, there were no differences in the mRNA expression of additional M1 (Tnf, Nos2) and M2 (Chil3, Arg1) markers by unprimed WT vs NG2KO BMDMs (M0), IFN-γ-primed WT vs NG2KO BMDMs (M1), or IL-4-primed WT vs NG2KO BMDMs (M2) (Fig. 7b). These results suggest that NG2 per se is not involved in the polarization process itself. However, obviously, studies on in vitro-cultured cells cannot exclude that the in vivo environment influences the polarization of macrophages in WT and NG2KO mice upon EAE induction.

### Both a modified immune response and a conserved BBB are responsible for the milder EAE manifestations in NG2KO mice

**Fig. 8:**
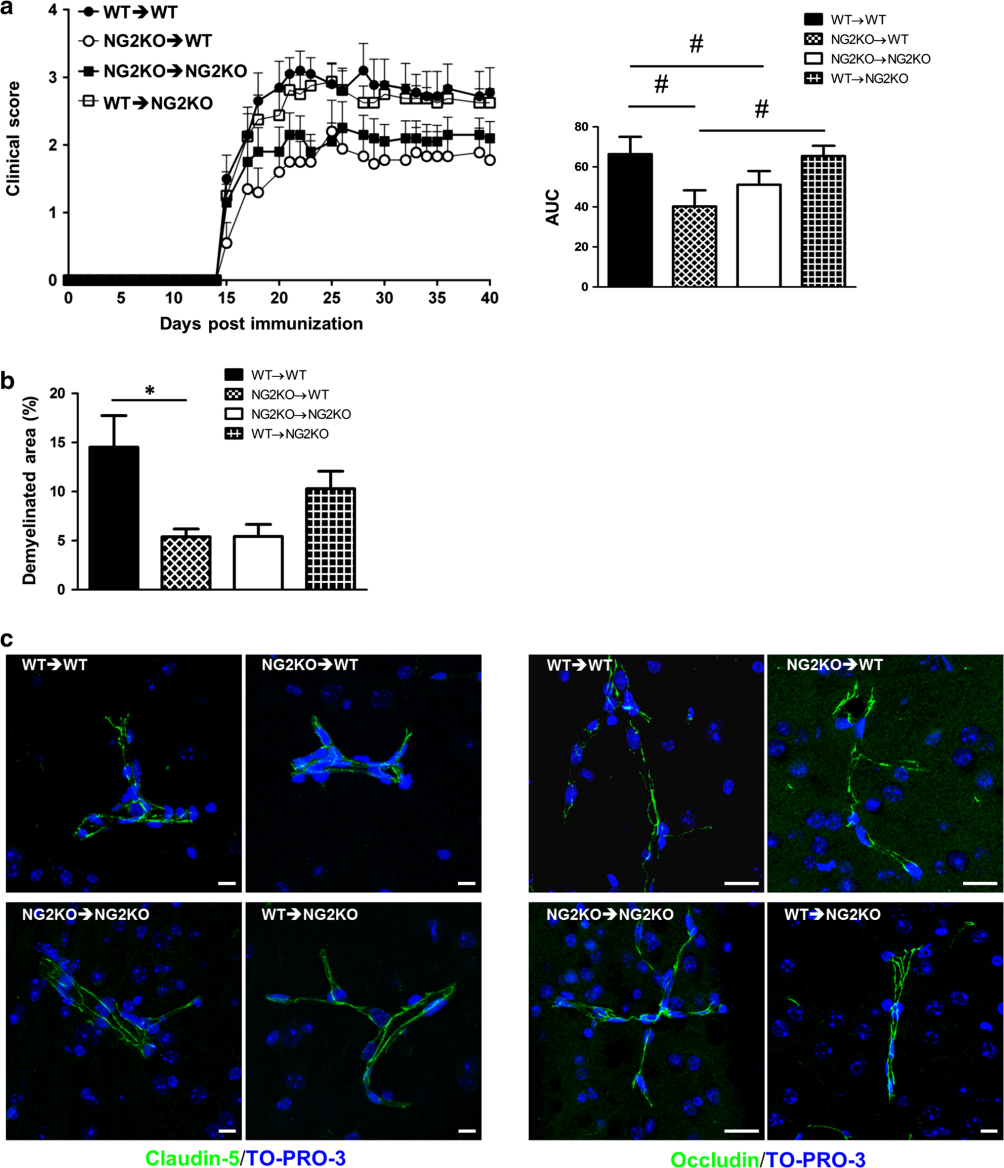
Lack of NG2 expression at both CNS/BBB and immune system levels contributes to the decrease in EAE severity observed in NG2KO mice. **a** Chimeric mice reconstituted with NG2KO bone-marrow cells develop a milder EAE, regardless of their inherent WT or NG2KO genotype. Data are shown as mean ± SEM daily clinical score (*left panel*) and mean ± SEM AUC of EAE clinical course calculated for each mouse (*right panel*). ^*#*^ *P* < 0.01. A representative of two independent experiments is presented (WT → WT, *n* = 10; NG2KO → WT, *n* = 10; NG2KO → NG2KO, *n* = 10; WT → NG2KO, *n* = 8). **b** Demyelination is decreased in CNS of NG2KO bone marrow recipients. Demyelinated areas were quantified by staining for myelin basic protein in spinal cord sections from WT → WT, NG2KO → WT, NG2KO → NG2KO, and WT → NG2KO EAE-affected mice (three sections per each of cervical, thoracic, and lumbar regions per mouse, *n* = 3–4 mice per group) sampled at chronic disease phase, 40 days post EAE induction. Data are expressed as mean ± SEM percent of demyelination. *P < 0.05. **c** Confocal microscopy images of cerebral cortex immunostained for claudin-5 and occludin in EAE-affected chimera mice at chronic disease stage (40 dpi) confirm the maintenance of the BBB features in NG2KO recipient chimera regardless of the donor immune cell phenotype. In EAE-affected WT recipient mice, reconstituted with WT donor (WT → WT) or NG2KO donor ((NG2KO → WT) bone marrow, the junctional staining, for both claudin-5 and occludin, is characterized by a discontinuous pattern with long, unstained tracts, whereas the NG2KO recipient counterparts (NG2KO → NG2KO and WT → NG2KO) show a continuous junctional staining. *Scale bars* 10 μm. Binary image rendering is shown in Supplementary Fig. S7

Altogether, the above findings suggested that the effect of NG2KO on the immune response might be of prime relevance in the attenuated clinical and pathological manifestations of EAE displayed by NG2KO mice. To ascertain this possibility, we generated chimeric mice reconstituted with whole bone-marrow cells, in which the role of the CNS milieu during EAE could be distinguished from that of the immune cells. Thus, WT NG2-sufficient mice were irradiated to eradicate the recipient immune cells and then reconstituted with NG2KO bone-marrow cells, and vice versa with irradiated NG2KO mice being transplanted with WT NG2-expressing bone-marrow cells. Control mice included irradiated WT mice receiving WT bone-marrow cells and irradiated NG2KO mice receiving NG2KO bone-marrow cells. Two months after transplantation, mice were immunized for EAE. As can be seen in Fig. 8a (left panel), while all groups developed disease at the same time, mice transplanted with NG2KO bone-marrow cells (NG2KO → WT and NG2KO → NG2KO) displayed lower clinical scores than mice transplanted with WT bone-marrow cells (WT → NG2KO and WT → WT); the clearly attenuated disease course in these groups was confirmed by statistical analysis of the AUC (Fig. 8a, right panel). The reduced clinical severity was accompanied by less severe neuropathology with reduced demyelination in recipients of NG2KO bone marrow (Fig. 8b). To analyze if reconstitution with the different immune systems had a differential effect on the BBB of the recipient mice, we analyzed the distribution of the endothelial TJ proteins, claudin-5 and occludin, in the chimera CNS microvessels. As shown in Fig. 8c, regardless of the donor bone marrow, the junctional staining patterns of EAE-affected WT recipient chimera were discontinuous, similar to that observed in EAE-affected WT mice (Fig. 3a), whereas in NG2KO recipient chimera interendothelial TJs appeared preserved as suggested by the continuous claudin-5/occludin staining pattern. These data indicate that the milder EAE observed in NG2KO mice results from both lower pathogenicity of NG2KO immune cells and a less leaky BBB in NG2KO recipients.

## Discussion

In this study, we demonstrate that NG2, a marker of OPCs and pericytes, whose expression on various immune cell types we demonstrate here for the first time, plays an important role in the induction and progression of EAE. We further demonstrate that this role exerts itself at both the CNS/BBB and immune system levels. Thus, in EAE, the lack of NG2 resulted in reduced loss of OPCs at the chronic stage of EAE and in preserved TJ continuity, hence reduced BBB leakage, at the CNS level, and in a skewed Th2 response likely due to a shift of stimulating DCs to a less inflammatory profile. Our findings contrast markedly with those obtained by Moransard et al., who reported that NG2 expressed by CNS-resident cells or by infiltrating macrophages did not play a role in EAE [35]. The reasons for this discrepancy are unclear as both studies use NG2KO mice derived from the original colony generated by Grako et al. [17]. While the EAE induction protocols in the two studies differ, with the protocol used by Moransard et al. comprising less *Mycobacterium tuberculosis* per injection but including a boost after 7 days [35], the severity and course of the disease in the WT mice are equivalent in both studies. One can only speculate that the divergence in results might be attributable to differences in the mice themselves. The NG2 protein deficiency in these mice was obtained by homologous recombination to create a loss-of-function mutation through disruption of the NG2 coding sequence via insertion of a neomycin-resistance fragment in the targeting vector [17]; it is, therefore, possible that mutation rescue might occur, with loss of the insertion [20]. In this context, it is interesting to note that Moransard et al. did observe some NG2 reactivity of CNS resident cells in their NG2KO chimera reconstituted with WT bone marrow [35]; unfortunately, they do not show the data for their NG2KO/NG2KO negative control chimera, nor do they show the confirmation of NG2 gene disruption in their NG2KO mice. We have verified the lack of NG2 in our NG2KO mouse colony through PCR analysis for the gene disruption (Supplementary Fig. S1) and through immunochemistry both at the CNS (OPCs and pericytes, Supplementary Fig. S3a and b) and immune cell (Supplementary Fig. S6) levels. Our data are supported by previous data obtained in the focal demyelination/remyelination model induced in mouse spinal cord by intrathecal injection of lysolecithin, where reduced inflammation was associated with less myelin damage and repair in NG2KO mice [28]. Indeed, in that study, naïve WT and NG2KO mice did not display differences in the numbers of PDGFRα^+^ OPCs, as we also observed; however, the acute demyelination stage (1 week after lysolecithin injection) was associated with lower numbers of OPCs and macrophages in NG2KO spinal cord, together with a significantly lower number of demyelinated axons, as compared to WT mice [28]. We also observed a significant reduction in demyelinated areas in spinal cord of EAE-affected NG2KO mice at 40 dpi, which was associated with decreased inflammation. We have reported that at the acute phase of EAE, there is a significant increase in OPC numbers in WT mice [16]; this does not occur in NG2KO mice, where the OPC numbers do not change throughout the disease. While this could relate to enhanced differentiation of NG2KO OPCs to pre-myelinating oligodendrocytes, it is more likely due to the reported decreased OPC proliferation in the absence of NG2 [28, 29], as the levels of GPR17^+^ PDGFRα^−^ cells paralleled those of OPCs in NG2KO mice throughout EAE, indicating that lack of NG2 is unlikely to alter OPC differentiation.

On par with the findings in lysolecithin-induced demyelination in NG2KO mice [28], we also observed a decreased expression of pro-inflammatory cytokines in CNS of EAE-affected NG2KO mice. These findings are further supported by our demonstration of the importance of NG2 in immune cell function, leading to a skewing of the T-cell response to the encephalitogenic peptide from a Th1 to a Th2 type. Kucharova et al. have suggested that NG2KO macrophages/microglia exhibit less inflammatory properties than their wild-type counterparts [28], a possibility supported by the in vitro demonstration that NG2KO microglia have a reduced inflammatory phenotype [13]. In vitro, Moransard et al. found that bone-marrow-derived macrophages, but not microglia, express low levels of NG2 that increase significantly upon exposure to TGF-β [35]. In vivo, several studies have detected NG2 expression on macrophage-like cells in the injured CNS [5, 26, 33], and Gao et al. observed colocalization of NG2 and OX42 (CD11b/c) immunoreactivity in the corpus callosum of rats injected with LPS which elicits the innate immune response [13]. Similarly, Jones et al., using a combination of IBA-1 immunolabeling and morphological analysis, identified macrophages, but not microglia, as cells producing NG2 after spinal cord injury [26]. In EAE, Moransard et al. suggest that there is overlap in macrophage distribution and NG2 immunoreactivity, albeit diffuse, in wild-type mice at the acute phase of the disease [35]. We have assessed the expression of NG2 on IBA-1^+^ macrophages/microglia in mice at 20 and 40 days after the encephalitogenic challenge and our results are in agreement with studies that showed the existence of a subpopulation of macrophage-like NG2^+^ cells in the CNS after insult. Indeed, while we never detected NG2^+^ IBA-1^+^ cells in EAE CNS at 40 dpi, we observed the presence of a scarce NG2^+^ macrophage-like population in the vicinity of EAE WT microvessels at 20 dpi (data not shown). In our hands, day 20 after immunization corresponds to the early chronic phase; it is, therefore, possible that we would have seen a greater number of NG2^+^ IBA-1^+^ cells at the acute phase, that is around days 10–15, as Moransard et al. report, albeit without defining the time frame of the acute phase in their EAE model [35].

We show that not only macrophages but also T cells and dendritic cells express NG2 and that its absence results in a modified function of these cells. To our knowledge, expression of NG2 on peripheral blood and bone-marrow cells has not been reported before in mice, and NG2^+^ cells were only found in blood of patients with special types of leukemia [38, 42, 43], using a monoclonal antibody, MoAb 7.1, raised against an SV-40-transformed human marrow stromal cell line, which recognizes a 220–240 kDa protein with a high degree of homology to rat NG2 [43]. It is not clear why NG2 should be expressed on normal immune cells in mice and not in humans, and we can only speculate that in humans NG2 expression on hematopoietic cells is aberrant and induced by the disease, as suggested [38], or that the different observation might be due to the difference in antibodies used. Indeed, in the present study we used a rabbit polyclonal antibody raised against purified rat NG2 and our FACS analysis of human peripheral blood cells from three control individuals with this polyclonal antibody shows that, in fact, human T cells, monocytes, and NK cells do express NG2 on their surface (Supplementary Fig. S8); it is, therefore, possible that the unique epitope recognized by MoAb 7.1 is masked in normal human blood cells and revealed only in modified human leukemic cells.

The potential role of NG2 expression on immune cells is unknown. Bu et al. suggest that its transient expression on macrophages invading the CNS upon excitotoxic hipoccampal lesioning is associated with an activated state of these cells [5]. However, we analyzed NG2 protein expression on non-activated cells freshly isolated from spleen or in vitro-derived from bone marrow and found that the majority of macrophages and T cells, and 50 % of dendritic cells express this molecule on their surface. As EAE is mediated by a T-cell response, mutations that affect T cells are likely to affect the T cell response and, therefore, the EAE course. In this context, we have observed a skewing of encephalitogen-reactive NG2KO T cells from a Th1 to a Th2 phenotype upon ex vivo recall response to MOG. Whether this is due to an intrinsic functional modification of NG2KO T cells or not is unclear. We have observed a strong upregulation of IL-4 mRNA expression in naïve NG2KO T cells as compared to WT T cells upon mitogenic stimulation, together with upregulation, albeit of a smaller extent, of IFN-γ and IL-17 mRNA (data not shown), suggesting that skewing towards a Th2 type could be an intrinsic feature of NG2KO T cells.

Nevertheless, it is likely that the skewing from Th1 to Th2 in MOG35-55-responsive NG2KO T cells is also related to a role of NG2 in DC activation. DCs are the main antigen-presenting cells in EAE [18], co-infiltrating the CNS with T cells [7]; their production of IL-12 is essential in the Th1-cell response implicated in the disease. IL-12 is a potent inducer of IFN-γ secretion by T cells; it drives the differentiation of naïve T cells to Th1 cells while impeding that to Th2 cells, and contributes to amplification and expansion of Th1 cells. In EAE, the role of IL-12 in disease expression is clear; thus, treatment with recombinant IL-12 increased disease severity [31, 44] and in vitro treatment of encephalitogen-reactive T cells with recombinant IL-12 enhanced their encephalitogenicity upon adoptive transfer EAE [31, 54]. We observed a reduction in IL-12 expression by stimulated NG2KO DCs at both mRNA and protein levels and the expression of IL-12-expressing CD11c^+^ cells was significantly decreased in MOG35-55-primed NG2KO lymph node cells; while this could be due to a decrease in migratory capacity of the DCs in NG2KO mice, possibly resulting from a stronger CD40 signaling [32], it could also indicate that IL-12 expression is governed by NG2 expression by the cells. Our results, which show that, in naïve WT mice, the proportion of DCs that express IL-12 is significantly lower in NG2^−^ than in NG2^+^ DCs, support this hypothesis. However, how NG2 expression influences the expression of IL-12 is unclear, and further studies that include silencing the NG2 gene in WT DCs are necessary to sustain this possibility.

Our data on the role of NG2 in immune cell function suggest that NG2 KO results in less inflammatory T cells and DCs; while this could explain the reduced inflammatory infiltration we see in NG2KO mice during EAE, the latter is also likely due to the lack of NG2^+^ pericytes and the maintained BBB in NG2KO mice during EAE. Thus, Stark et al. showed that NG2^+^, but not NG2^−^, pericytes interact dynamically with extravasated innate immune cells to promote their migration along capillaries and arterioles [48]. Our observation of a maintained BBB in naïve NG2KO mice and its reinforced integrity during EAE was unexpected. Several studies on tumor vascularization, where NG2 expression is upregulated in activated pericytes, have indicated that lack of NG2 results in reduced pericyte/endothelial cell interaction and basal lamina assembly with increased vessel leakiness [15, 25, 58]. However, CNS interendothelial TJs and BBB functionality had not been studied in healthy NG2KO mice until now. In our study, naïve NG2KO mice were viable, healthy, fertile, and indistinguishable from WT mice, indicating that NG2 was not essential in BBB-microvessel development. Nevertheless, the modified appearance of the junctional staining pattern described in naïve NG2KO mice implies that NG2 is involved in the TJ settling plan and that its lack during brain development could lead to subtler alterations of claudin-5 and occludin distribution. As revealed by freeze-fracture electron microscopy, TJ-associated integral membrane proteins form linear chains of particles, denoted TJ strands, which are arranged in regular meshes and correspond to focal attachments of the affronted junctional membranes [45, 46, 51]. When considered in the context of strand organization, the aspect of naïve NG2KO TJs, upon claudin-5 and occludin immunostaining, may represent a reduced number of strands/meshes in the belt between two adjoining endothelial cells (bicellular TJs) and apparently normal points of intersections between three joining endothelial cells (tricellular TJs). The reason why vessel development is negatively impacted in tumors in the absence of NG2 may depend on a substantially different organization as compared to CNS microvessels. Thus, whereas tumor capillaries are built up from two cell types, pericytes and endothelial cells, the neurovascular unit typical of CNS microvessels is assembled from both cellular and non-cellular components, with the cellular component also including perivascular astrocytes that are thought to induce the barrier phenotype of endothelial cells [1, 8, 21]. Unlike that observed for NG2-expressing macrophages in tumors, whose role prevails on NG2^+^ pericyte activity in determining vascular abnormalities (Yotsumoto et al. 2015), the results obtained through the analysis of EAE-affected NG2KO mouse chimeras confirm that TJ organization in CNS microvessels is primarily controlled by perivascular cells, astrocyte and pericytes. Thus, chimera of NG2KO mice reconstituted with WT bone marrow and induced for EAE did not show increased leakiness as compared to their NG2KO counterparts reconstituted with NG2KO bone marrow.

In conclusion, we show that the lack of NG2 in NG2KO mice impacts EAE through its effect not only at the CNS level, more particularly at the BBB, but also most importantly at the immune response level. Our data further suggest that NG2, expressed on immune cells, might be involved in the activation of DCs and thereby of the encephalitogenic T-cell response, through controlling IL-12 expression.

## Electronic supplementary material

Below is the link to the electronic supplementary material.

Supplementary material 1 (JPEG 809 kb). Supplementary Fig. S1 Genotyping analysis confirms disruption of the gene encoding NG2 in NG2KO mice. Genomic DNA samples extracted from tails of WT and NG2KO mice 30 days after birth were analyzed by PCR using Cspg4 primers (5’-CGCTGACCTCCGATGTTC-3’ and 5’-AAGTTGCCACGCTTGTCC-3’) that amplify a 200 bp sequence of exon 3 from nucleotides 637 to 838 in wild-type mice. Gene disruption in the NG2KO mice results from the insertion of a 1100-bp neomycin-resistance fragment in exon 3 at nucleotide 672, which leads to a loss-of-function mutation. Electrophoresis of the amplified DNA from WT and NG2KO mice on 1% agarose gel shows the expected 200 bp band in the WT sample and 1300-bp band in each of the four NG2KO samples

Supplementary material 2 (JPEG 1722 kb). Supplementary Fig. S2 During EAE, microglia/macrophages are reduced in NG2KO CNS. Confocal microscopy images of microglia/macrophages (IBA-1, red) and microvessel basal lamina (Coll IV, green) in cerebral cortex (a) and spinal cord (b) of naïve and EAE-affected (40 dpi) WT and NG2KO mice. IBA-1+ microglia/macrophages cells in EAE-affected WT brain, unlike those from naïve WT brain that display a resting dendritic shape, appear activated and characterized by an intermediate phenotype (arrows) or atypical round amoeboid morphology (arrowheads). Nuclei are stained with TO-PRO-3 (blue). Scale bar: 10 m

Supplementary material 3 (JPEG 2053 kb). Supplementary Fig. S3 Confirmation of NG2 ablation in OPCs and pericytes, and validation of alternative marker in morphometric analysis for quantification of OPCs in CNS. OPC numbers remain constant throughout EAE in NG2KO spinal cord. a Confocal microscopy confirms the co-localization of NG2 and PDGFRα in naïve WT OPCs (left panel): NG2 is detected predominantly on the OPC processes (arrow), whereas PDGFRα appears more concentrated on the cell body and proximal processes (arrowhead); in naïve NG2KO mice (right panel), OPCs stain only for PDGFRα (arrowheads). b In EAE-affected WT mice (20 dpi), immunostaining for NG2 is observed on activated pericytes stained concomitantly for CD13 (arrow), as well as on OPC ramified branches (left panel); in EAE-affected NG2KO mice (20 dpi), as expected, NG2 reactivity is absent and pericytes appear only labeled by CD13 (arrows) (right panel). Nuclei are stained with TO-PRO 3 (blue). (C) Quantification of PDGFRα+ OPCs in naïve, EAE-affected (20 and 40 dpi) WT and NG2KO mice. 3 sections were taken at 50 m intervals from each of the cervical, thoracic and lumbar regions of the spinal cord (n = 5 mice per group); data are presented as mean ± SD cell number/mm3, #P< 0.05. Scale bars: a, 30 m; b, 5 m

Supplementary material 4 (JPEG 494 kb). Supplementary Fig. S4 Altered distribution of tight-junction proteins in naïve and EAE-affected NG2KO spinal cord parallels that of cerebral cortex. Representative confocal microscopy images of claudin-5 and occludin expression in spinal cord of naïve and EAE-affected NG2KO mice (40 dpi). As for cerebral cortex, an irregular pattern of claudin-5 and occludin reactivity along the endothelial edges is seen in naïve mice (arrows), whereas in EAE-affected NG2KO mice the strong junctional immunostaining is continuous along the endothelial profile (arrowheads). Nuclei are stained with TO-PRO-3 (blue). Scale bars: 20 m and 10 m, for claudin-5 and occludin images, respectively

Supplementary material 5 (JPEG 214 kb). Supplementary Fig. S5 As expected, NG2 is not detected on NG2KO immune cells. Immunostaining of NG2KO splenocytes for NG2 (green) and CD11b (red) confirms the lack of expression of NG2 on NG2KO immune cells. Nuclei are stained with DAPI (blue). Scale bar: 5 μm. Magnification x100

Supplementary material 6 (JPEG 185 kb). Supplementary Fig. S6 MOG35-55 T-cell lines generated from WT and NG2KO mice do not differ in their cytokine profile. MOG35-55-specific T-cell lines were generated from primed lymph node cells and maintained as previously described [35]. The concentration of indicated cytokines was measured by ELISA on culture supernatants from T-cell proliferation to MOG35-55 performed in the presence of irradiated splenocytes as antigen-presenting cells as previously described [35]

Supplementary material 7 (JPEG 446 kb). Supplementary Fig. S7 Binary image rendering of the data presented in Fig. 8c.

Supplementary material 8 (JPEG 997 kb). Supplementary Fig. S8 NG2 is also expressed on human immune cells. Peripheral blood mononuclear cells (PBMCs) were obtained from blood samples of three healthy control (HC) volunteers at three independent times and prepared over Ficoll density gradient according to the manufacturer’s instructions (Cedarlane, Burlington, Canada). PBMCs (1 x 106) were double-stained with anti-NG2 Alexa Fluor®488 antibody (diluted 1:33, Merck) and antibodies against surface markers, PercP-conjugated anti-CD3 (diluted, 1:33, Biolegend), APC Cy7-conjugated anti-CD14 (diluted, 1:33, Biolegend) and PE Cy7-conjugated anti-CD56 (diluted, 1:33, Biolegend) antibodies, for T cells, monocytes, and natural killer (NK) cells, respectively. PBMCs were analyzed by flow cytometry using a FACS Canto II Calibur (Becton Dickinson). a Dot plots of HC1, HC2 and HC3 show the concomitant expression of NG2 and CD3 in T cells, NG2 and CD14 in monocytes, and NG2 and CD56 in NK cells among human PBMCs. Red: single NG2 reactivity; purple: CD3+ cells; blue, CD14+ cells; green, CD56+ cells. As can be seen (red dots), not all PBMCs stained for NG2, and some PBMCs that did not stain for CD3, CD14 or CD56 expressed NG2. b Summary data from the three different HCs confirms that the majority of human T cells, monocytes and NK cells express NG2. Quantitative data are presented as mean ± SEM. c Assessment of mean fluorescence intensity (MFI) of NG2 expression by cells from the three different HCs revealed that the expression of NG2 is highest on NK cells, followed by monocytes, and lowest on T cells
