# Supplementary figures and images for: NG2, a common denominator for neuroinflammation, blood–brain barrier alteration, and oligodendrocyte precursor response in EAE, plays a role in dendritic cell activation
Source: Acta Neuropathol. 2016 Mar 30;132:23–42. doi: 10.1007/s00401-016-1563-z (PMC4911384; doi:10.1007/s00401-016-1563-z)

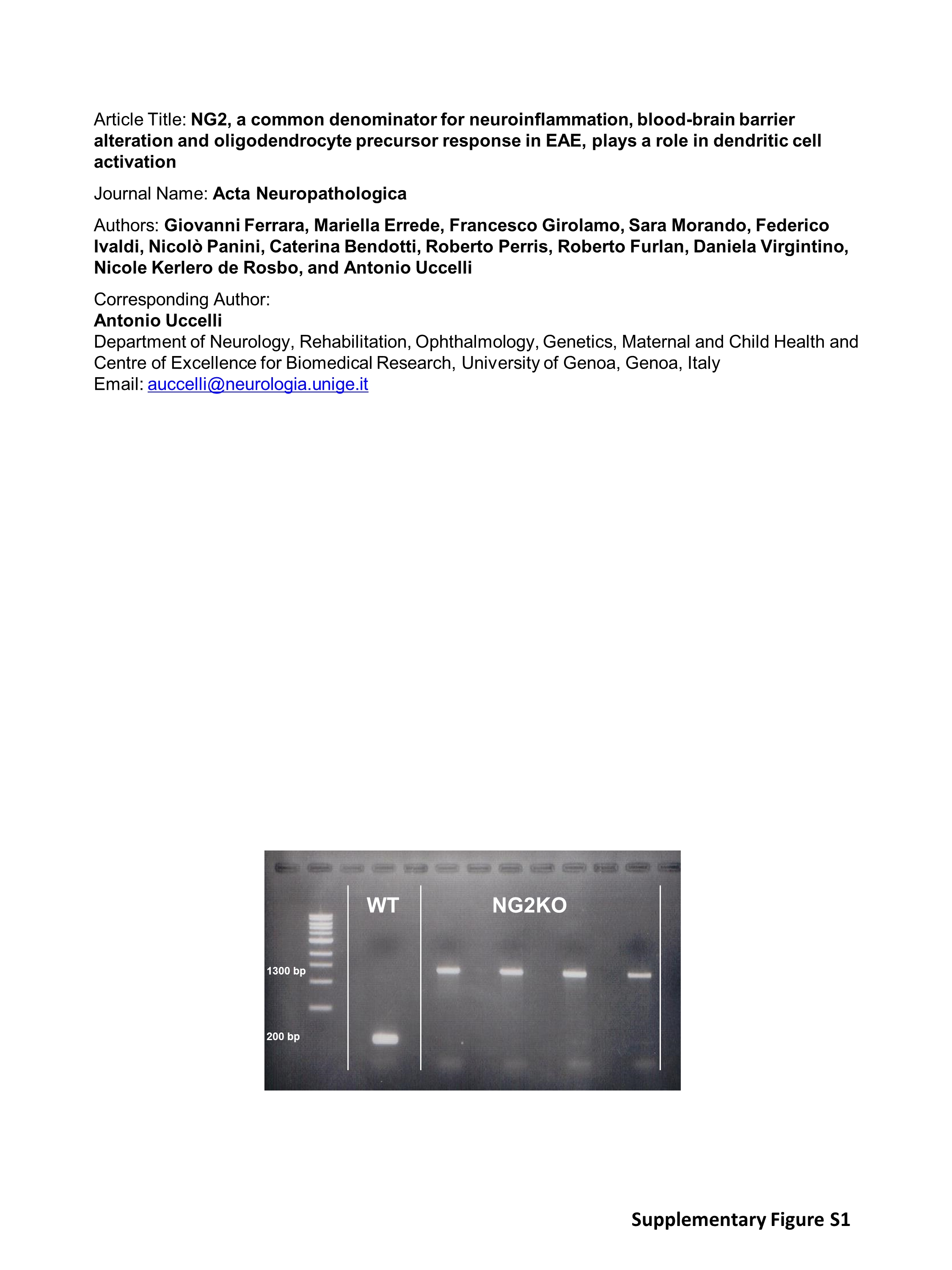

Supplement: Supplementary file 1 — Supplementary material 1 (JPEG 809 kb). Supplementary Fig. S1 Genotyping analysis confirms disruption of the gene encoding NG2 in NG2KO mice. Genomic DNA samples extracted from tails of WT and NG2KO mice 30 days after birth were analyzed by PCR using Cspg4 primers (5’-CGCTGACCTCCGATGTTC-3’ and 5’-AAGTTGCCACGCTTGTCC-3’) that amplify a 200 bp sequence of exon 3 from nucleotides 637 to 838 in wild-type mice. Gene disruption in the NG2KO mice results from the insertion of a 1100-bp neomycin-resistance fragment in exon 3 at nucleotide 672, which leads to a loss-of-function mutation. Electrophoresis of the amplified DNA from WT and NG2KO mice on 1% agarose gel shows the expected 200 bp band in the WT sample and 1300-bp band in each of the four NG2KO samples [file 401_2016_1563_MOESM1_ESM.jpg]

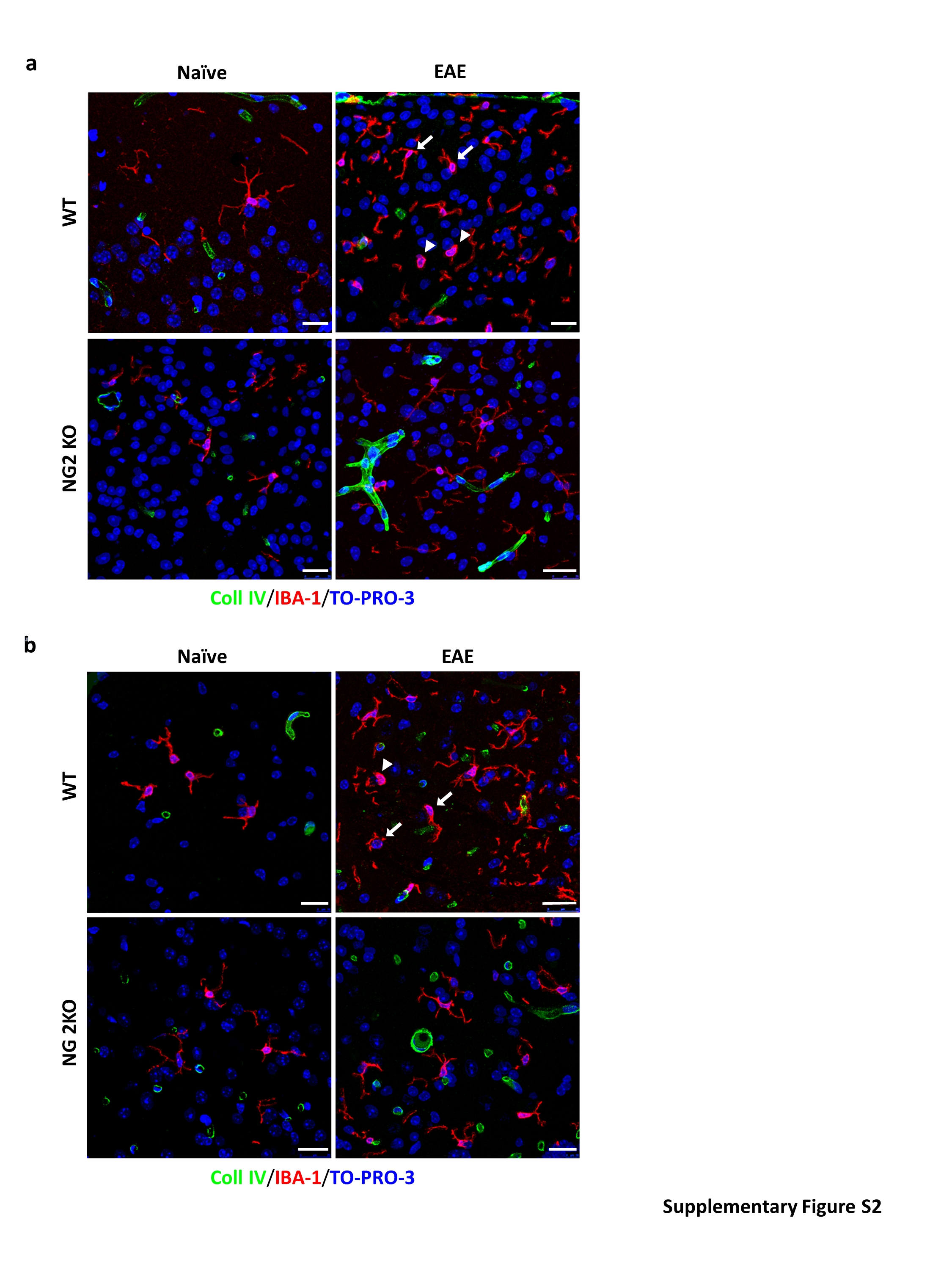

Supplement: Supplementary file 2 — Supplementary material 2 (JPEG 1722 kb). Supplementary Fig. S2 During EAE, microglia/macrophages are reduced in NG2KO CNS. Confocal microscopy images of microglia/macrophages (IBA-1, red) and microvessel basal lamina (Coll IV, green) in cerebral cortex (a) and spinal cord (b) of naïve and EAE-affected (40 dpi) WT and NG2KO mice. IBA-1+ microglia/macrophages cells in EAE-affected WT brain, unlike those from naïve WT brain that display a resting dendritic shape, appear activated and characterized by an intermediate phenotype (arrows) or atypical round amoeboid morphology (arrowheads). Nuclei are stained with TO-PRO-3 (blue). Scale bar: 10 m [file 401_2016_1563_MOESM2_ESM.jpg]

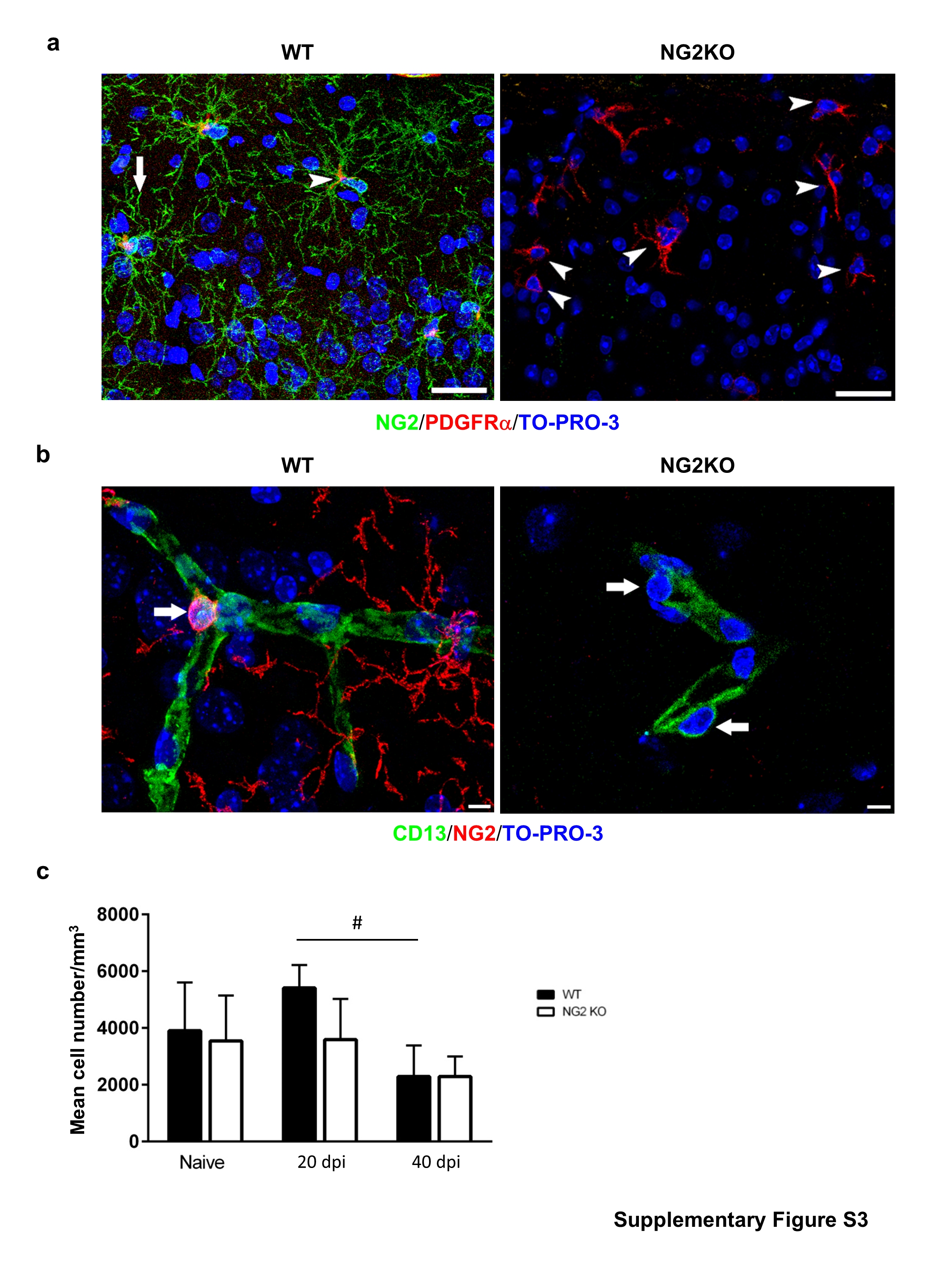

Supplement: Supplementary file 3 — Supplementary material 3 (JPEG 2053 kb). Supplementary Fig. S3 Confirmation of NG2 ablation in OPCs and pericytes, and validation of alternative marker in morphometric analysis for quantification of OPCs in CNS. OPC numbers remain constant throughout EAE in NG2KO spinal cord. a Confocal microscopy confirms the co-localization of NG2 and PDGFRα in naïve WT OPCs (left panel): NG2 is detected predominantly on the OPC processes (arrow), whereas PDGFRα appears more concentrated on the cell body and proximal processes (arrowhead); in naïve NG2KO mice (right panel), OPCs stain only for PDGFRα (arrowheads). b In EAE-affected WT mice (20 dpi), immunostaining for NG2 is observed on activated pericytes stained concomitantly for CD13 (arrow), as well as on OPC ramified branches (left panel); in EAE-affected NG2KO mice (20 dpi), as expected, NG2 reactivity is absent and pericytes appear only labeled by CD13 (arrows) (right panel). Nuclei are stained with TO-PRO 3 (blue). (C) Quantification of PDGFRα+ OPCs in naïve, EAE-affected (20 and 40 dpi) WT and NG2KO mice. 3 sections were taken at 50 m intervals from each of the cervical, thoracic and lumbar regions of the spinal cord (n = 5 mice per group); data are presented as mean ± SD cell number/mm3, #P< 0.05. Scale bars: a, 30 m; b, 5 m [file 401_2016_1563_MOESM3_ESM.jpg]

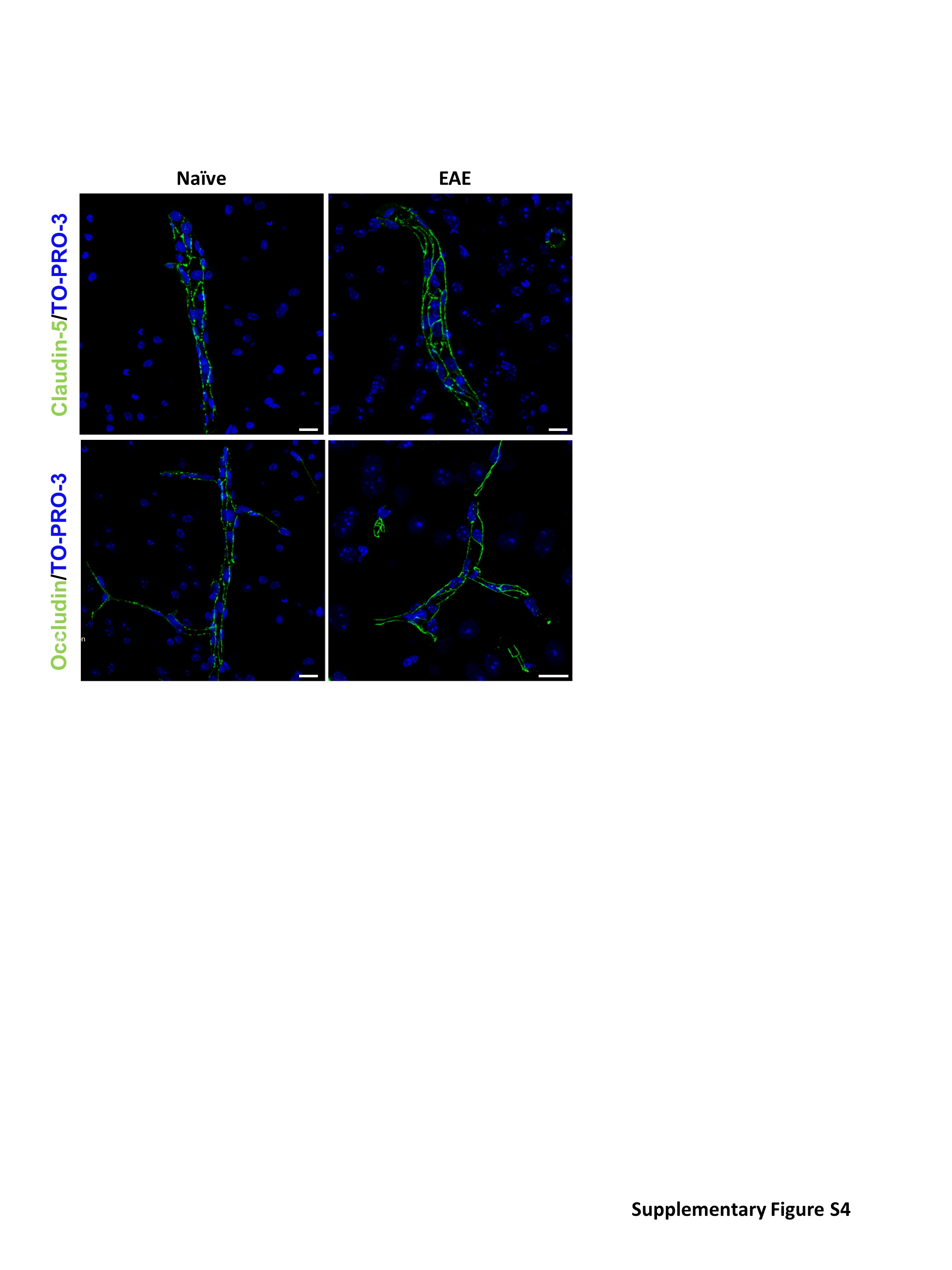

Supplement: Supplementary file 4 — Supplementary material 4 (JPEG 494 kb). Supplementary Fig. S4 Altered distribution of tight-junction proteins in naïve and EAE-affected NG2KO spinal cord parallels that of cerebral cortex. Representative confocal microscopy images of claudin-5 and occludin expression in spinal cord of naïve and EAE-affected NG2KO mice (40 dpi). As for cerebral cortex, an irregular pattern of claudin-5 and occludin reactivity along the endothelial edges is seen in naïve mice (arrows), whereas in EAE-affected NG2KO mice the strong junctional immunostaining is continuous along the endothelial profile (arrowheads). Nuclei are stained with TO-PRO-3 (blue). Scale bars: 20 m and 10 m, for claudin-5 and occludin images, respectively [file 401_2016_1563_MOESM4_ESM.jpg]

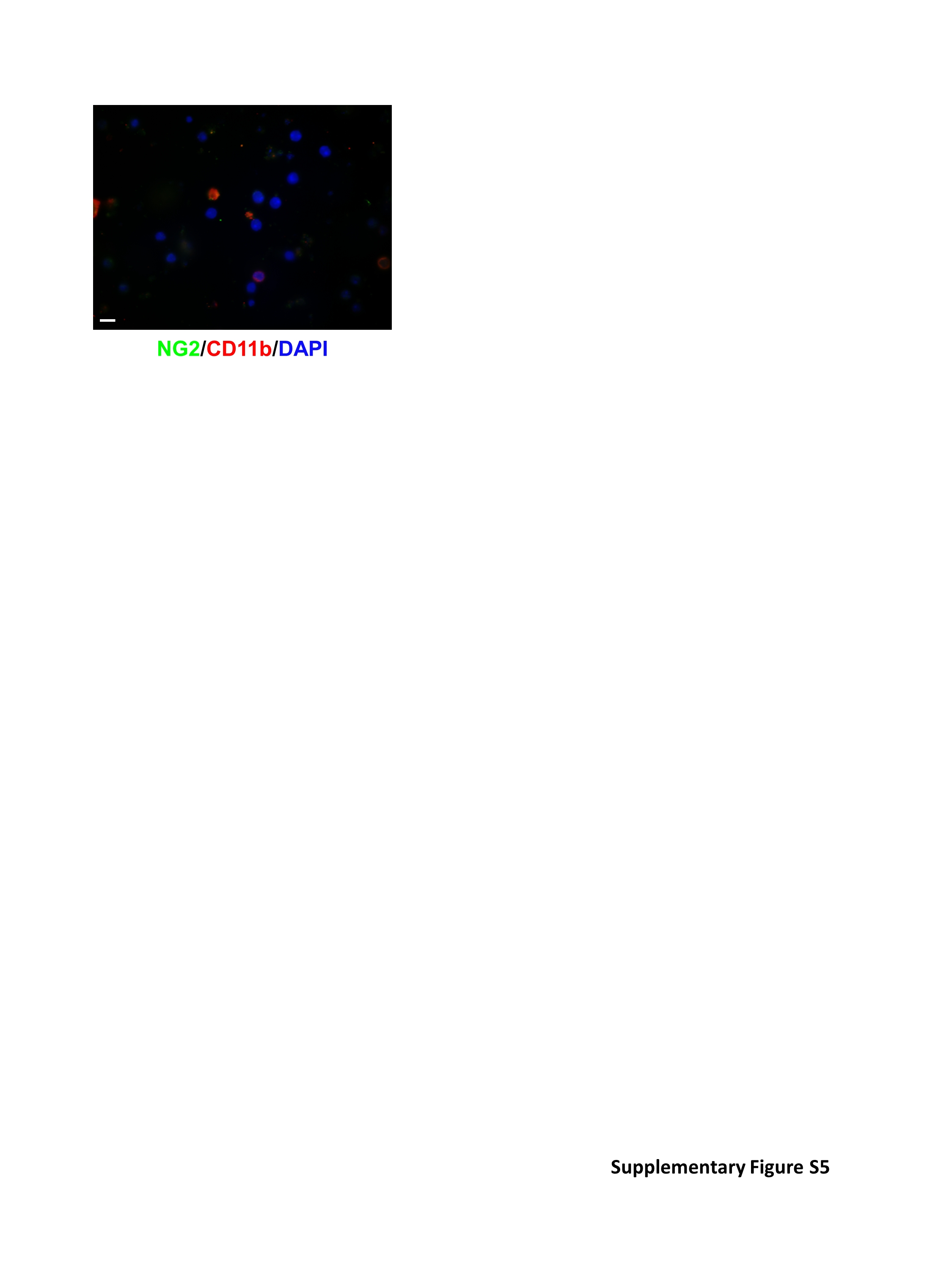

Supplement: Supplementary file 5 — Supplementary material 5 (JPEG 214 kb). Supplementary Fig. S5 As expected, NG2 is not detected on NG2KO immune cells. Immunostaining of NG2KO splenocytes for NG2 (green) and CD11b (red) confirms the lack of expression of NG2 on NG2KO immune cells. Nuclei are stained with DAPI (blue). Scale bar: 5 μm. Magnification x100 [file 401_2016_1563_MOESM5_ESM.jpg]

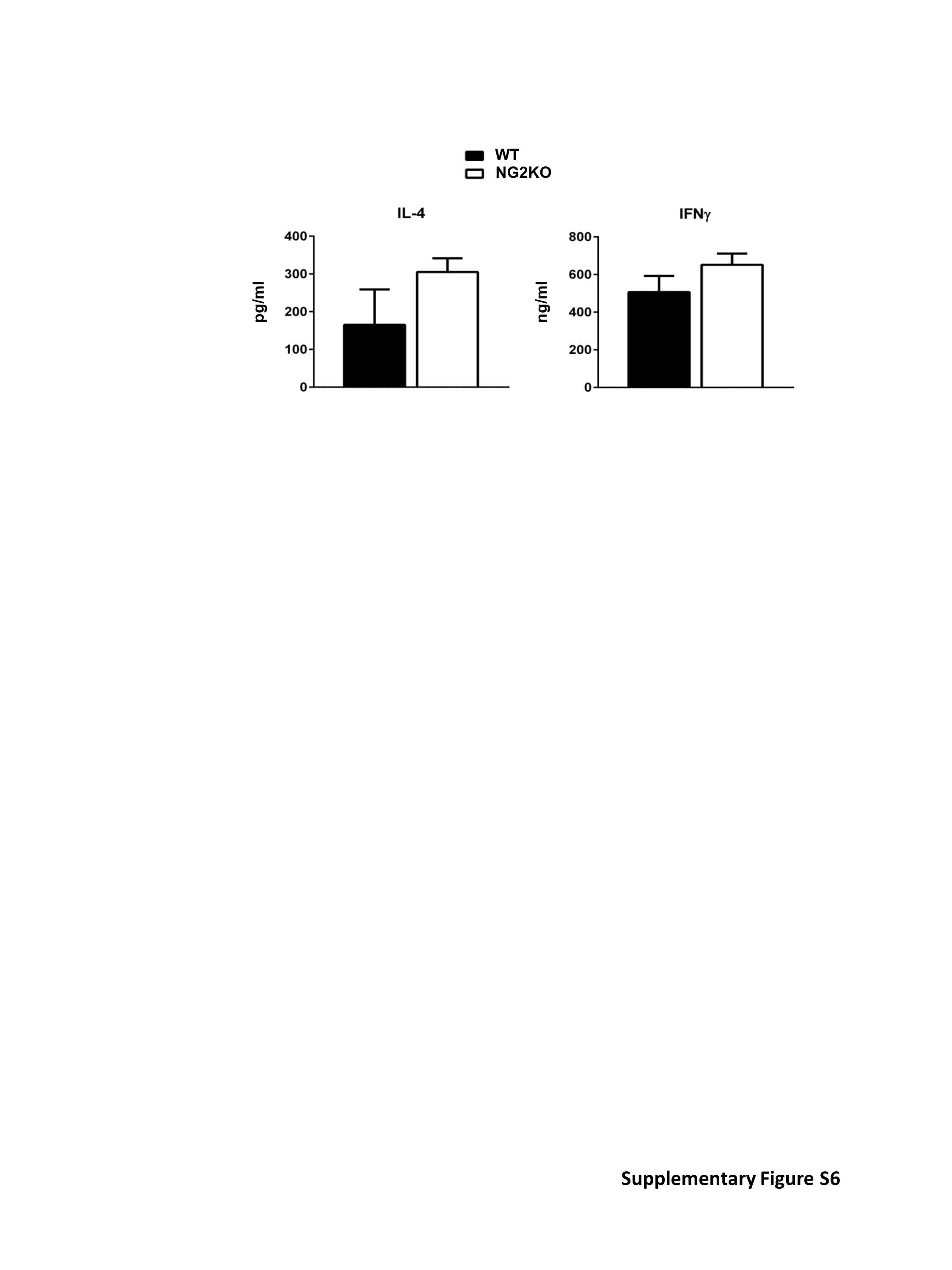

Supplement: Supplementary file 6 — Supplementary material 6 (JPEG 185 kb). Supplementary Fig. S6 MOG35-55 T-cell lines generated from WT and NG2KO mice do not differ in their cytokine profile. MOG35-55-specific T-cell lines were generated from primed lymph node cells and maintained as previously described [35]. The concentration of indicated cytokines was measured by ELISA on culture supernatants from T-cell proliferation to MOG35-55 performed in the presence of irradiated splenocytes as antigen-presenting cells as previously described [35] [file 401_2016_1563_MOESM6_ESM.jpg]

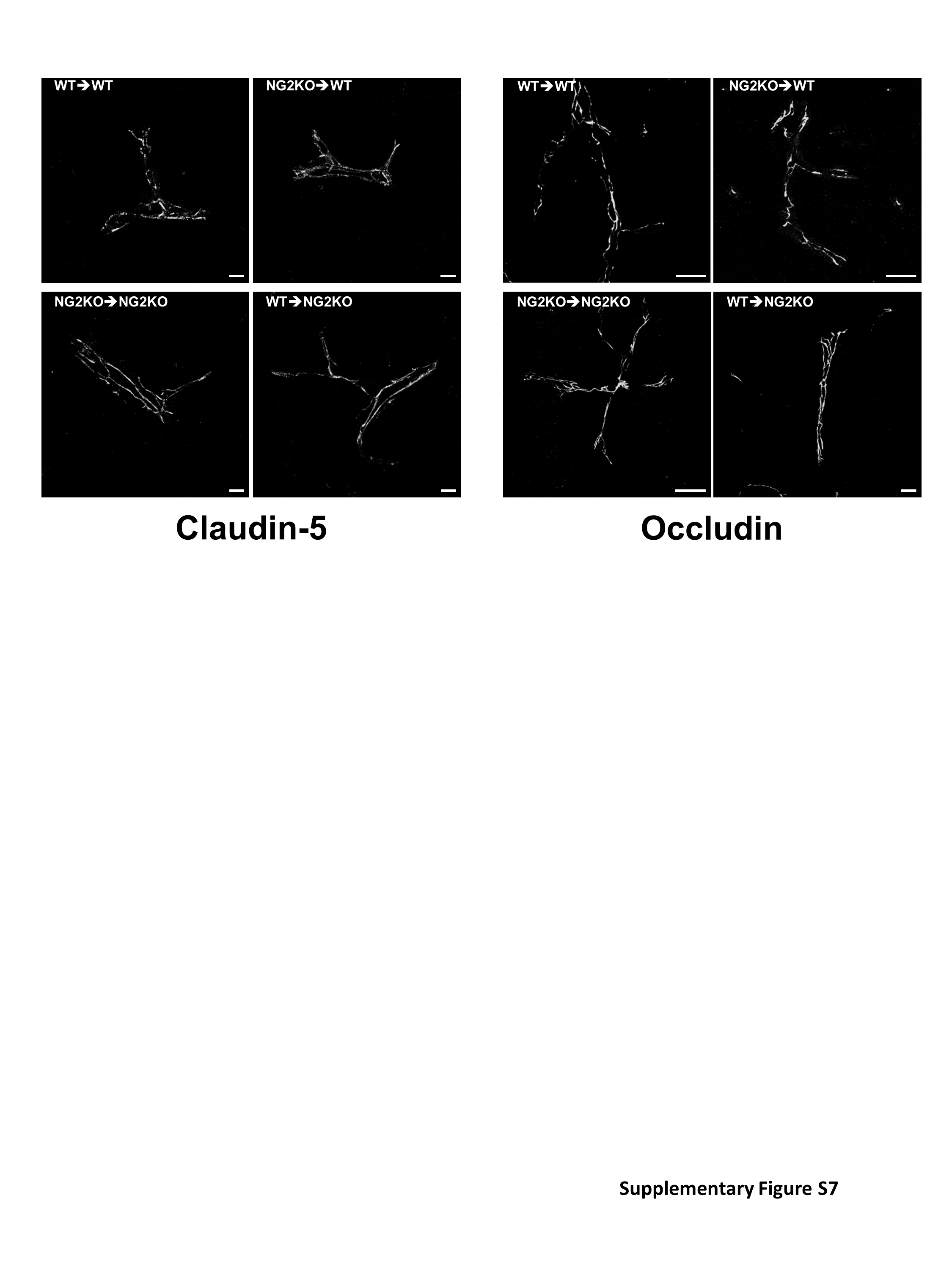

Supplement: Supplementary file 7 — Supplementary material 7 (JPEG 446 kb). Supplementary Fig. S7 Binary image rendering of the data presented in Fig. 8c. [file 401_2016_1563_MOESM7_ESM.jpg]

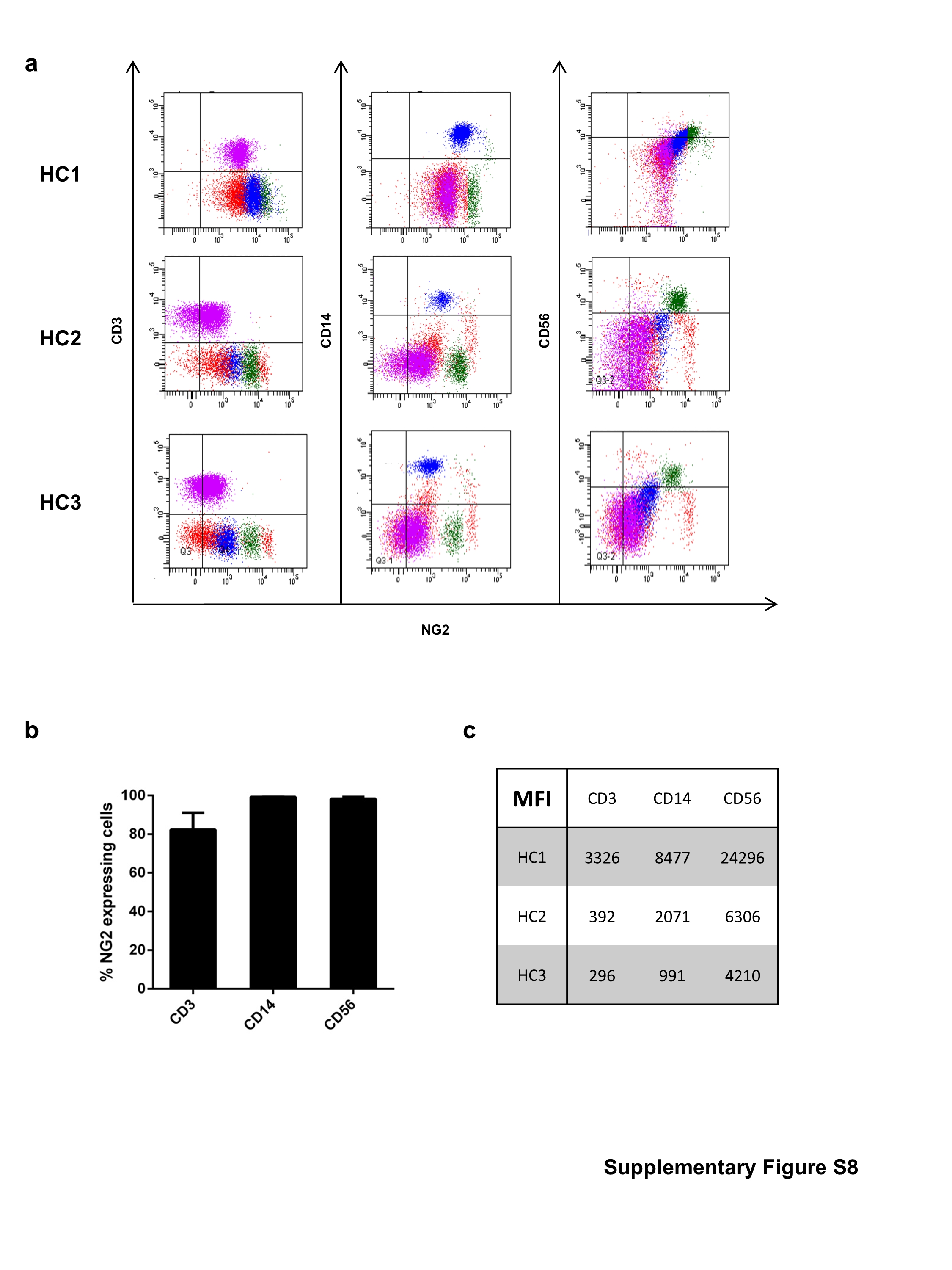

Supplement: Supplementary file 8 — Supplementary material 8 (JPEG 997 kb). Supplementary Fig. S8 NG2 is also expressed on human immune cells. Peripheral blood mononuclear cells (PBMCs) were obtained from blood samples of three healthy control (HC) volunteers at three independent times and prepared over Ficoll density gradient according to the manufacturer’s instructions (Cedarlane, Burlington, Canada). PBMCs (1 x 10^6^) were double-stained with anti-NG2 Alexa Fluor®488 antibody (diluted 1:33, Merck) and antibodies against surface markers, PercP-conjugated anti-CD3 (diluted, 1:33, Biolegend), APC Cy7-conjugated anti-CD14 (diluted, 1:33, Biolegend) and PE Cy7-conjugated anti-CD56 (diluted, 1:33, Biolegend) antibodies, for T cells, monocytes, and natural killer (NK) cells, respectively. PBMCs were analyzed by flow cytometry using a FACS Canto II Calibur (Becton Dickinson). a Dot plots of HC1, HC2 and HC3 show the concomitant expression of NG2 and CD3 in T cells, NG2 and CD14 in monocytes, and NG2 and CD56 in NK cells among human PBMCs. Red: single NG2 reactivity; purple: CD3+ cells; blue, CD14+ cells; green, CD56+ cells. As can be seen (red dots), not all PBMCs stained for NG2, and some PBMCs that did not stain for CD3, CD14 or CD56 expressed NG2. b Summary data from the three different HCs confirms that the majority of human T cells, monocytes and NK cells express NG2. Quantitative data are presented as mean ± SEM. c Assessment of mean fluorescence intensity (MFI) of NG2 expression by cells from the three different HCs revealed that the expression of NG2 is highest on NK cells, followed by monocytes, and lowest on T cells [file 401_2016_1563_MOESM8_ESM.jpg]
